# Optimizing pediatric “Mild” traumatic brain injury assessments: A multi-domain random forest analysis of diagnosis and outcomes

**DOI:** 10.1016/j.ijchp.2025.100600

**Published:** 2025-06-27

**Authors:** Upasana Nathaniel, Erik B. Erhardt, Divyasree Sasi Kumar, Jingshu Wu, Samuel D. Miller, Pawani Chauhan, Rahsan Keskin, Tracey V. Wick, Keith Owen Yeates, Timothy B. Meier, Harm J. van der Horn, John P. Phillips, Richard A. Campbell, Robert E. Sapien, Andrew R. Mayer

**Affiliations:** a The Mind Research Network/Lovelace Biomedical Research Institute, Albuquerque, NM 87106, USA; b Department of Mathematics and Statistics, University of New Mexico, Albuquerque, NM 87131, USA; c Department of Physical Medicine & Rehabilitation, University of New Mexico, Albuquerque, NM 87131, , USA; d Department of Psychology, University of Calgary, Calgary, Alberta, Canada; e Alberta Children's Hospital Research Institute, University of Calgary, Calgary, Alberta, Canada; f Hotchkiss Brain Institute, University of Calgary, Calgary, Alberta, Canada; g Neurosurgery, Medical College of Wisconsin, Milwaukee, WI 53226, USA; h Cell Biology, Neurobiology and Anatomy, Medical College of Wisconsin, Milwaukee, WI 53226, USA; i Biomedical Engineering, Medical College of Wisconsin, Milwaukee, WI 53226, USA; j University of Groningen, University Medical Center Groningen, Groningen, the Netherlands; k Department of Neurology, University of New Mexico, Albuquerque, NM 87131, USA; l Department of Psychiatry & Behavioral Sciences, University of New Mexico, Albuquerque, NM 87131; m Department of Emergency Medicine, University of New Mexico, Albuquerque, NM 87131, USA; n Department of Psychology, University of New Mexico, Albuquerque, NM 87131, USA

**Keywords:** Mild traumatic brain injury, Pediatric, Clinical assessments, Diagnostic classification, Outcome classification, Machine learning

## Abstract

**Objective:**

Despite advances in imaging and fluid-based biomarkers, the care for pediatric “mild” traumatic brain injury (pmTBI) remains primarily dependent on clinical evaluation. However, the optimal clinical assessments for diagnosing pmTBI and predicting outcomes remain debated, including which individual test or combinations of assessments are most effective, and how this evolves as a function of time post-injury.

**Method:**

Random Forest models were used to identify the most effective assessments for diagnostic (pmTBI vs. healthy controls) and outcome (pmTBI with favorable vs. poor outcomes, based on persisting symptoms) classification accuracy across a comprehensive battery including domains of self-reported clinical-ratings, paper-and-pencil cognitive tests, computerized cognitive tests, symptom provocation during neurosensory tests, and performance-based neurosensory measures. Assessments were conducted within 11-days, at 4-months and 1-year post-injury to examine acute and long-term recovery trajectories. A total of 323 pmTBI (180 males; age 14.5 ± 2.8 years) and 244 HC (134 males, 14.0 ± 2.9 years) were included (∼75 % 1-year retention) in final analyses.

**Results:**

Self-reported clinical-ratings outperformed performance-based metrics across all visits in both models, with somatic complaints demonstrating the highest predictive validity. Cognitive tests of memory aided diagnostic classification, while emotional disturbances were predictive of outcome classification up-to 4-months. Retrospective ratings, reflecting trait-like characteristics, were more predictive for identifying individuals at risk of poor outcomes. Computerized cognitive and neurosensory tests had limited predictive value beyond 1-week post-injury.

**Conclusions:**

Clinicians should adopt a tailored approach for clinical assessments across different post-injury intervals to enhance clinical care, shorten assessment batteries, and better understand recovery in children with “mild” TBI.

## Introduction

Pediatric “mild” traumatic brain injury (pmTBI), synonymously referred to as concussion (Silverberg et al., 2023), impacts over 1000,000 children annually in the United States alone, placing a substantial burden on both children and their families (Bryan et al., 2016; Taylor et al., 2017). While most children recover (Ledoux et al., 2019; McCarthy et al., 2006), a notable minority (∼15–35 %) experiences short and long-term challenges associated with their injury (Yeates et al., 2019; Zemek et al., 2016). These challenges often manifest as various post-concussion symptoms (PCS), which may include somatic complaints (e.g., headaches, nausea, dizziness, etc.), cognitive difficulties (e.g., memory and executive function), neurosensory disturbance (e.g., ocular, vestibular, and auditory), emotional distress (e.g., anxiety, depression), or behavioral problems (Polinder et al., 2018; Robertson-Benta et al., 2023). These difficulties can significantly affect interpersonal functioning, well-being, scholastic performance, and overall quality of life (van Ierssel et al., 2021).

Despite recent advancements in using imaging and blood-based biomarkers to classify TBI severity (Maas et al., 2022), diagnosis and determination of outcome status following pmTBI still primarily relies on clinical evaluations. These clinical evaluations can be broadly defined as belonging to two different classes. The first class includes measures of subjective experiences, most traditionally through self- and/or parent-reports of PCS and related conditions such as anxiety and depression (Yeates et al., 2023). More recently, there has been an effort to measure changes in self-reported subjective experiences during various challenges, hereafter referred to as symptom provocation (Corwin et al., 2014; Mayer et al., 2018; Mayer, Wertz et al., 2020; Mucha et al., 2014; Sufrinko et al., 2017), including following exercise, as a benchmark for concussion recovery (Leddy et al., 2019). The second set of clinical evaluations can be broadly defined as performance-based measurements (e.g., errors, reaction time, convergence distance, etc.) of cognitive and neurosensory functioning (Mayer et al., 2018). Performance-based assessments were traditionally performed with paper-and-pencil tests but have more recently shifted toward computerized versions (Sicard et al., 2022).

However, debate remains about which specific clinical evaluations, or combination of evaluations, are the most effective in diagnosing pmTBI and are associated with poor versus favorable outcomes (Polinder et al., 2018), and how these evaluations may evolve as a function of time post-injury. While most children demonstrate post-concussive deficits acutely, research remains inconclusive on whether these disturbances extend significantly over time post-injury relative to typically developing children (Beauchamp et al., 2018; Moore et al., 2018; Sicard et al., 2019). Recovery trajectories are highly variable following pmTBI as a result of complex interactions between injury characteristics (e.g., severity, age at injury, and post-concussive symptoms), host (e.g., sex, pre-morbid cognitive and behavioral functioning), and environmental (e.g., socioeconomic status, parent mental health) factors (Tuerk et al., 2020). As a result, recent research has called for a paradigm shift in how we assess and manage persisting symptoms after concussion (PSaC), advocating for a more integrative, biopsychosocial approach that considers the individual variability in recovery and the multiple domains that can be affected (Broshek et al., 2022).

Machine learning has the potential to address challenges associated with determining the optimal composition of clinical evaluations by enabling multi-dimensional interactions between variables and identifying and ranking the most critical measures for detecting deficits at various times post-injury (Yim et al., 2020). Random Forest, an ensemble method based on decision trees, has shown significant promise in handling complex neuropsychological data and offers excellent predictive performance for diagnoses (Battista et al., 2017; Graham et al., 2020). Studies comparing various algorithms, such as logistic regression, decision trees, and support vector machine, have found Random Forest to be the most effective for optimizing cognitive evaluations and enhancing diagnostic accuracy (Wang et al., 2022). The Random Forest feature selection method identifies the degree to which each feature is important for predicting diagnostic and outcome determination, both independent of and conditional on the other features, which can further help simplify assessment batteries and ultimately reduce clinical burden. Collinearity does not adversely affect the quality of prediction, unlike for statistical inference, where collinearity adversely inflates standard errors of estimates and p-values.

The current study therefore used Random Forest analyses to identify individual assessments (i.e., features) from within five separate domains (self-reported clinical-ratings, traditional paper-and-pencil cognitive tests, computerized cognitive tests, symptom provocation following neurosensory measures, and performance-based measures on neurosensory tests) that exhibited the best accuracy for diagnostic (pmTBI vs. healthy controls) and outcome (pmTBI with favorable vs. poor outcomes, based on presence or absence of PSaC) classification. “Mild” TBI was classified using published criteria (see methods) to maintain continuity with existing publications while simultaneously recognizing that the nomenclature of “mild” may not accurately reflect underlying injury conditions (Maas et al., 2022; Manley et al., 2025; Silverberg et al., 2023). These analyses were conducted at 3 different post-injury intervals (i.e., within 11 days of injury and at approximately 4 months and 1-year post-injury) to better understand both acute and long-term recovery trajectories. The top performers from each individual domain were subsequently entered into a final model to determine the best-performing features for diagnostic and outcome classification at each visit.

## Materials and methods

### Participants

**Fig. 1:**
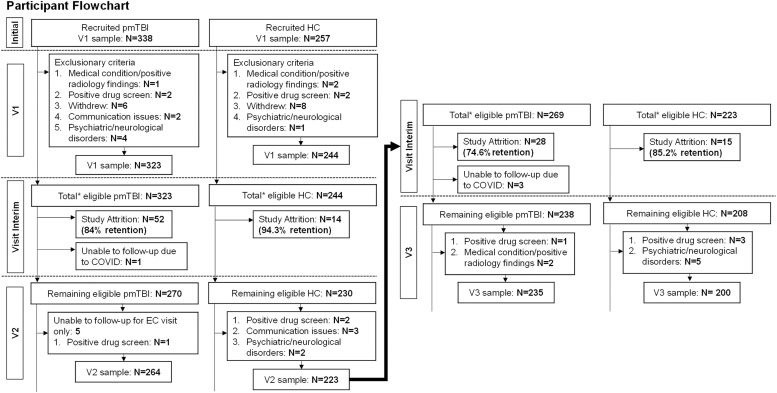
Participant recruitment and retention. Flowchart of enrolment, inclusion and data quality control from Visits V1 (=within 11 days of injury), V2 (approximately 4-months post-injury) and V3 (approximately 1-year post-injury) for patients with pediatric “mild” traumatic brain injury (pmTBI) and matched healthy controls (HC). The asterisk denotes the total number of participants who were eligible to return, which is a sum of participants with usable clinical data and those whose data was excluded at previous visits due to quality assurance issues.

Pediatric mTBI patients (*N* = 338, Fig. 1) aged 8–18 years were consecutively recruited from local emergency and urgent care departments from July 2016 to December 2023 and diagnosed by clinicians independent of the study. Inclusion criteria for pmTBI consisted of 1) a closed head injury with a Glasgow Coma Score (GCS) ≥ 13, 2) loss of consciousness (LOC) <30 min, 3) post-traumatic amnesia (PTA) <24 h, 4) an alteration in mental status, or 5) at least two new acute symptoms within 48 h of injury. Criteria were therefore a blend of the American Congress of Rehabilitation Medicine and Zurich Concussion in Sport Group criteria. Follow-up assessments occurred within 11 days of injury (V1) and at approximately 4 months (V2) and 1-year post-injury (V3). The 4-month time point is critical, as many individuals with pmTBI remain clinically symptomatic at this stage. Similarly, the 1-year time point helps assess whether symptoms persisting beyond 3 months, which are indicative of chronic changes, continue to impact functioning and long-term outcomes (Yeates et al., 2004). Age- and sex-matched (*N* = 257, Fig. 1) typically developing healthy children (HC) were recruited from the local community through fliers and by word-of-mouth. HC were assessed at equivalent timepoints to account for potential neurodevelopmental confounds or effects associated with repeat assessment.

Exclusion criteria for both pmTBI and HC included 1) history of neurological diagnoses, 2) history of moderate or severe TBI (LOC greater than 30 min), 3) severe developmental disorders (autism spectrum disorder or intellectual disability), 4) psychiatric disorders other than adjustment disorder, 5) substance abuse/dependence, or 6) non-English fluency. Finally, all participants underwent urine screening at all three visits to rule out substance abuse/dependence, with positive screens resulting in exclusion from the study except for recreational marijuana use. Additional exclusion criteria for HC were a history of attention-deficit/hyperactivity disorder or a learning disability. Participants with pmTBI were further excluded if injury affected the dominant hand, or if general anesthesia was administered during routine trauma care. The University of New Mexico Health Sciences Institutional Review Board approved all procedures. Per institutional guidelines, both participants and parents provided informed consent (ages 12–18) or assent (ages 8–11).

### Procedures

A comprehensive clinical battery was completed by all participants and their parents at each study visit (see Table 1). Participants and parents independently completed age-/rater-appropriate (see Supplemental Materials for modifications) versions of the Post-Concussion Symptom Inventory (PCS), Conflict and Behavioral Questionnaire (Behavior), and Pediatric Quality of Life Inventory (QoL) at each visit. A retrospective rating of symptoms one month before the initial visit was also performed (V1 only). Participants additionally completed retrospective and concurrent Patient-Reported Outcomes Measurement Information System (sleep, anxiety, and depression), self-reported pain, and headache ratings. Patients with pmTBI were binarily classified as having PSaC separately for each visit, based on standardized scores generated from the HC using V1 data only (Mayer, Stephenson et al., 2020; see Supplemental Materials). The presence (i.e., poor) versus absence (i.e., favorable) of PSaC was subsequently used to stratify pmTBI based on outcome. Previous history of mTBI was assessed through a semi-structured interview (Hergert et al., 2022). Finally, a modified 5P risk score (Zemek et al., 2016) was calculated based on available demographic and clinical data (see Supplemental Materials).

**Table 1:** Primary and secondary clinical and cognitive measures.

Instrument	Measured domain	Rater	Visit
**Demographics**			
NewMAP TBI	Self-reported TBI history	C & P	R, V1, V2, V3
Tanner Stage of Development	Pubertal development	C	V1, V2 & V3
BSI-18 (Symp)	Parental psychopathology	P	V1, V2 & V3
**Clinical Domain**			
PCSI (PCS)	Post-concussive symptoms	C; P	R, V1, V2 & V3
PROMIS Sleep (SLP)	Sleep disturbance	C	R, V1, V2 & V3
PROMIS Anxiety (AX)	Anxiety symptoms	C	R, V1, V2 & V3
PROMIS Depression (DP)	Depressive symptoms	C	R, V1, V2 & V3
Pain scale (PN)	Pain	C	R, V1, V2 & V3
HIT-6 (HD)	Headache functional impact	C	R, V1, V2 & V3
CBQ (BH)	Behavior	C; P	R, V1, V2 & V3
SDQ (PA)	Behavioral screening for psychological attributes	P	R, V2 & V3
PedsQL (QoL)	Health-related quality of life	C; P	R, V2 & V3
**Cognitive Domain**			
DKEFS Color-Word interference Stroop Color Naming (C), Word Reading (R), Inhibition (I); Cogstate Identification task (IDN)	Attention	C	V1, V2 & V3
WAIS-IV/WISC-V Coding and Symbol Search; Cogstate Detection task (DET)	Processing speed	C	V1, V2 & V3
WISC-V/WAIS-IV Digit Span Backwards; Cogstate One-Back task (ONB)	Working memory	C	V1, V2 & V3
HVLT-R Immediate Recall; Cogstate One-Card Learning (OCL)	Visual learning/memory	C	V1, V2 & V3
DKEFS Trail Making Test Number Sequence (Trails A), Number Letter Sequence (Trails B), Verbal Fluency, Color-Word interference Stroop Inhibition/Switching (IS)	Executive function	C	V1, V2 & V3
HVLT-R Delayed Recall	Long-Term Memory Recall	C	V1, V2 & V3
**Neurosensory Domain**			
Double Dorsal Foot Stretch (DDFS)	Non-neurosensory symptom validity	C	V1, V2 & V3
Smooth Pursuit (Sm Pur)	Vestibular-ocular and ocular motor functions	C	V1, V2 & V3
Horizontal Saccades (H Sac)		C	V1, V2 & V3
Vertical Saccades (V Sac)		C	V1, V2 & V3
Horizontal VOR (H VOR)		C	V1, V2 & V3
Vertical VOR (V VOR)		C	V1, V2 & V3
Visual Motion Sensitivity (VMS)		C	V1, V2 & V3
Near Point Convergence (NPC)	Visual convergence insufficiency	C	V1, V2 & V3
Monocular Accommodation (MA)	Accommodative dysfunction	C	V1, V2 & V3
Tandem Gait (TG)	Balance	C	V1, V2 & V3
King-Devick (KD)	Saccadic eye movements	C	V1, V2 & V3

Notes: Instrument– NewMAP TBI: New Mexico Assessment of Pediatric TBI, BSI: Brief Symptom Inventory-18, PCSI: Post-Concussion Symptom Inventory, PROMIS: Patient-Reported Outcomes Measurement Information System, HIT-6: Headache Impact Test, CBQ: Conflict Behavior Questionnaire, SDQ: Strengths and Difficulties Questionnaire, PedsQL: Pediatric Quality of Life Inventory, DKEFS: Delis-Kaplan Executive Function System, WAIS-IV: Wechsler Adult Intelligence Scale–IV, WISC-V: Wechsler Intelligence Scale for Children–V, HVLT-R: Hopkins Verbal Learning Test Revised; Rater—C: child, P: parent; V: Visit; R: retrospective.

A paper-and-pencil cognitive battery (see Table 1 and Supplemental Materials) included selected tests from the Delis-Kaplan Executive Function System (Stroop Color Naming, Word Reading, Inhibition, Inhibition/Switching; Trail Making Number Sequence, Number Letter Sequence; and Letter Fluency), the Hopkins Verbal Learning Test-Revised (HVLT-R Immediate and Delayed Recall), and the Digit Span Backwards, Symbol Coding, and Symbol Search subtests from either the Wechsler Adult Intelligence Scale-IV (WAIS-IV) or the Wechsler Intelligence Scale for Children-V (WISC-V), depending on age at enrollment. Participants also completed a computerized cognitive test (Cogstate), which measured attention/vigilance (identification task), processing speed (detection task), visual learning (one-card learning), and executive functioning (one-back task).

A comprehensive neurosensory battery was administered to assess symptom provocation as well as neurosensory performance (Mayer et al., 2018). This battery included the smooth pursuit, horizontal and vertical saccades, horizontal and vertical vestibular-ocular reflexes, and visual motion sensitivity subtests from the Vestibular/Ocular Motor Screening (VOMS). Participants also completed a 20-second dorsiflexion of the bilateral feet for a non-neurosensory control measure of symptom provocation (Mayer et al., 2018). The Astron accommodative rule (Gulden Ophthalmics, Elkins Park, PA) was used to assess near point of convergence, as well as left and right monocular accommodative amplitude. Participants completed a tandem gait task in which they were instructed to take 5 steps forward and 5 steps backward, with their eyes open and closed, along a 15-foot taped line. Errors were quantified as any loss of balance that resulted in a step off the tape. Finally, the King-Devick test, which measures rapid number naming, was electronically administered with response time and errors recorded. Symptom provocation was quantified following each neurosensory assessment based on the VOMS methodology. Specifically, participants rated 4 symptoms (headache, dizziness, nausea, and fogginess) on an 11-point Likert scale both prior to the neurosensory assessment (baseline) and after administering each sub-test. The change in total score from baseline for each subtest was quantified as the symptom provocation score (Elbin et al., 2022).

### Analytic plan

Chi-square tests and Generalized Linear Models were used to compare demographics across HC and pmTBI using SPSS version 20. The primary purpose of the current study was to determine diagnostic and outcome classification accuracy rather than statistical differences between group means. Random Forest analyses were therefore performed using the “randomForestSRC” (3.3.1) package with function “rfsrc” (Ishwaran & Kogalur, 2021) and 200 trees in the R software platform (4.4.1, R Core Team, 2023). Random Forest is a supervised ensemble learning algorithm in which multiple classification trees (a “forest”) are fit on bootstrapped samples (Breiman, 2001; Ishwaran & Malley, 2014). Each tree partitions selected features into a random subset of predictor variables to optimally maximize classification accuracy. Distributional assumptions are minimal, and external cross-validation is established by predicting group membership based on trees estimated from subsamples. Random Forest is robust to feature collinearity, and the bootstrap aggregating technique minimizes data overfitting. Prediction error rate plots confirmed that all models converged, and a minimum of 150 iterations were used during bootstrapping to determine mean variable importance scores (VIMP) and establish reliable VIMP confidence intervals. The mean VIMP quantifies the change in percent classification accuracy when that feature is excluded from the model, whereas the confidence interval establishes whether a feature reliably contributed to higher classification accuracy across the bootstrapped samples (Ishwaran & Kogalur, 2021). Thus, VIMP scores represent the primary metric for determining feature selection in Random Forest models. Feature selection for advancement to the final testing round was determined solely based on the lower bound of the 90 % VIMP confidence interval, which was required to be greater than 0. Setting this threshold means that the feature reliably contributed to classification accuracy in 95 % of resamples.

Separate Random Forest models evaluated both diagnostic (pmTBI vs. HC) and outcome (pmTBI with favorable vs. poor outcome based on presence or absence of PSaC) classification accuracy at each study visit. All available measures (see Table 1) were first separated into 5 individual domains and were analyzed within each domain. This was followed by final diagnostic and outcome Random Forest analyses that directly compared the best-performing features across all domains at each visit. Individual domain corresponded to 1) self-reported clinical-ratings (retrospective and current reports), 2) traditional paper-and-pencil neuropsychological tests, 3) computerized cognitive tests (Cogstate), 4) symptom provocation scores during neurosensory testing, and 5) performance-based measures on neurosensory tests. An additional domain corresponding to injury severity characteristics (5P risk score, mechanism of injury, presence/absence of LOC/PTA, and number of previous mTBI) was also included for evaluating outcome classification accuracy only based on previously published results (Yeates et al., 2009).

A subset of pmTBI participants (*N* = 155) underwent computed tomography (CT) scans as part of routine care, with 13 pmTBI participants (8.4 %) diagnosed with a positive CT finding by a physician independent of the study. Findings included skull fractures (*n* = 4), subarachnoid hemorrhage (*n* = 8), cerebral contusions (*n* = 2), and subdural hematomas (*n* = 3), with some participants presenting with multiple findings. Due to the low number of participants with positive CT results, this feature was not included in the outcome analyses.

Receiver Operating Characteristic (ROC) and the area under the ROC curve (AUC) were used to evaluate each model’s ability to distinguish between diagnostic groups or outcome. ROC curves, which plot sensitivity (true positive rate) and specificity (false positive rate), provide a visual representation of a model’s ability to discriminate between classes, while balanced accuracy offers a metric that equally weighs both sensitivity and specificity. The AUC of the ROC curve provides a summary index of model performance, overall decision thresholds, and reflects the overall predictive value of the features. Overall model performance was therefore characterized as weak (0.51–0.60), moderate (0.61–0.70), or good (>0.71) based on the AUC for each domain using published criteria (de Hond et al., 2022; Pencina et al., 2012). Similarly, the degree of collinearity between features in each domain was classified using Pearson’s correlation coefficient (r) separately for pmTBI and HC at each study visit. Minimal (*r* < 0.40), moderate (*r* = 0.40–0.69), or high (*r* > 0.70) collinearity was assessed using published criteria (Overholser & Sowinski, 2008). Note, however, that feature collinearity does not adversely affect model prediction accuracy.

## Results

### Demographics

Final analyses (See Fig. 1) included a total of 323 pmTBI (180 males; age 14.5 ± 2.8 years; 7.3 ± 2.2 days post-injury) and 244 HC (134 males, 14.0 ± 2.9 years) at V1. A total of 264 pmTBI (147 males; age 14.3 ± 2.8 years; 133.1 ± 19.1 days post-injury; 125.7 ± 18.9 days between V1 and V2) and 223 HC (126 males, 13.9 ± 2.8 years; 128.1 ± 19.1 days between V1 and V2) completed V2. Attrition occurred for 52 pmTBI (84 % retention) and 14 HC (94.3 % retention) between V1 and V2. A total of 235 pmTBI (130 males; age 14.2 ± 2.7 years; 372.6 ± 32.1 days post-injury; 366.8 ± 29.6 days between V2 and V3) and 200 HC (116 males, 13.7 ± 2.8 years; 369.2 ± 30.2 days between V2 and V3) completed V3. Retention rates for V3 were 74.6 % of recruited pmTBI and 85.2 % of recruited HC. All available data were used in analyses, including data from participants who did not complete all visits. See Supplemental Materials for full details on enrollment and retention rates across both cohorts, as well as socio-economic status and race (Table S2).

The pmTBI and HC groups did not differ in biological sex, age, self-reported Tanner stage of development, or handedness (all *p*’s≥0.05; see Table 2). Compared to the HC group, the pmTBI group self-reported a history of previous head injuries more often (*χ^2^*=22.20, *p* < 0.001; pmTBI=18.3 %; HC=6 %) and their parents reported worse overall psychopathology (*Wald-χ^2^*=28.81, *p* < 0.001). There were no significant differences (*p* > 0.05) for demographics and PCSI ratings at V1 between returning and non-returning participants.

**Table 2:** Demographics and injury characteristic data.

	V1 pmTBI (*N* = 323)	V1 HC (*N* = 244)
Age	14.8(12.7–16.8)	14.0(12.0–16.4)
Sex ( % Female)	44.3 %	45.1 %
Tanner Stage of Development	4(3–4)	4(2–4)
Parent BSI-18^a^	3(1–7)	1(0–5)
mTBI History^a^	18.3 %	5.8 %
**Injury Characteristics**		
Loss of Consciousness	49.8 %	–
Post-Traumatic Amnesia	38.1 %	–
**Mechanism of Injury**		
Struck by Object	14.6 %	–
Struck by Person	28.2 %	–
Fall	18.9 %	–
MVC	28.8 %	–
Assault	4.3 %	–
Bicycle-related	4.0 %	–
Other	0.6 %	–
**Sport or Recreation Related**	56.7 %	–

Notes: HC=healthy control; pmTBI=pediatric “mild” traumatic brain injury; BSI=Brief Symptom Inventory-18; MVC=motor vehicle crash. Data are formatted at median (interquartile range) based on distribution properties. *^a^*= Group main effect.

### Collinearity within each domain

Metrics of collinearity (Pearson r) were determined separately for each group at each visit within our 5 primary domains (see Supplemental Results and Tables S3-S7). Moderate collinearity (shown by 58.1 % of all unique measurement pairs at V1; 48.3 % at V2; 42.5 % at V3) existed for HC within the clinical-ratings domain (see Table S3), with strong collinearity between current and retrospective clinical-ratings measures at V1, reflecting more consistent ratings when assessments were conducted in close temporal proximity. A similar pattern of moderate collinearity (37.1 % at V1; 43.3 % at V2; 36.7 % at V3) was present for pmTBI, although with fewer strong correlations between retrospective and current ratings at V1 (anxiety, depression, and behavior only). Pediatric mTBI also showed strong negative correlations between quality of life and ratings of anxiety and depression at later visits.

Neurosensory symptom provocation measures showed a high proportion of strong (34.6 % at V1; 61.8 % at V2; 50.9 % at V3) as well as moderate (50.9 % at V1; 27.3 % at V2; 32.7 % at V3) collinearity for HC (see Table S4). Pediatric mTBI also demonstrated numerous strong (21.8 % at V1, 29.1 % at V2 and 43.6 % at V3) and moderate (76.4 % at V1, 52.7 % at V2, 34.6 % at V3) correlations between symptom provocation measures. The high degree of collinearity was likely influenced by zero inflation in both groups. In contrast, performance-based measures from the neurosensory battery (see Table S5) demonstrated minimal evidence of collinearity across all 3 visits for both groups.

Paper-and-pencil cognitive tests (see Table S6) showed moderate collinearity for HC (21.8 % at V1; 21.8 % at V2; 25.6 % at V3) and pmTBI (28.2 % at V1; 19.2 % at V2; 23.1 % at V3) across all visits, with strong positive correlations existing mostly within individual tests (e.g., HVLT-R and Stroop subtests). Similarly, moderate or strong collinearity existed on Cogstate computerized cognitive assessments (see Table S7) primarily for response time measures, which varied in a test-dependent fashion for both HC (moderate collinearity: 25 % at V1; 21.4 % at V2; 21.4 % at V3) and pmTBI (moderate collinearity: 32.1 % at V1; 21.4 % at V2; 21.4 % at V3). Specifically, similar to previous findings (Miyake et al., 2000), a strong correlation existed between identification and detection tasks, and one-card learning and one-back tasks.

### Diagnostic classification

#### Visit 1 (Approximately 1-week post-injury)

Random Forest analyses using V1 data demonstrated that all retrospective and current self-reported clinical-ratings, except for current behavior, improved classification accuracy based on our VIMP inclusion criterion (i.e., lower bound of the 90 % VIMP confidence interval > 0), resulting in good overall classification rates for this domain based on the AUC (see Table 3). Similar findings emerged for the neurosensory symptom provocation model, with the majority of neurosensory tests being selected based on VIMP scores during the Random Forest analysis, yielding moderate classification accuracy (see Table 4) despite relatively high collinearity. In contrast, for performance-based neurosensory measures, only the King-Devick Test (reaction time) met VIMP criteria for feature selection, with a moderate AUC (see Table 4). A similar level of moderate classification accuracy was observed for paper-and-pencil cognitive tests, with immediate and delayed recall (HVLT-R) and Stroop inhibition selected as features (see Table 5). In contrast, no features were selected for the computerized testing domain for diagnostic classification.

**Table 3:** Variable Importance score (VIMP %) for the diagnostic and outcome models for the clinical-ratings domain: Retrospective and Current child ratings.

Clinical Domain								
Metric		Tests	DX			Outcome		
			V1	V2	V3	V1	V2	V3
Child ratings: Feature selection	Retrospective	Post-Concussion Symptoms	1.19*	0.53	0.31	3.69*	4.12*	2.54
		Sleep	2.24*	2.85*	2.48*	2.28*	1.33	2.78*
		Pain	2.09*	0.50	0.74	0.22	0.78	1.09
		Headache	1.67*	2.84*	2.99*	1.32*	0.95	1.35
		Anxiety	1.57*	0.38	0.46	0.96	1.69	1.07
		Depression	1.16*	0.71	0.25	3.66*	0.35	0.25
		Behavior	1.07*	0.08	0.18	0.27	1.03	0.47
		Quality of Life	1.31*	0.40	0.88	3.26*	2.20	3.41*
	Current	Post-Concussion Symptoms	8.56*	2.41*	0.30	NA	NA	NA
		Sleep	3.89*	4.20*	2.34*	4.13*	2.75*	6.07*
		Pain	11.24*	0.72	−0.02	13.38*	3.36	1.05
		Headache	6.53*	1.95*	1.59	9.50*	4.55*	4.68*
		Anxiety	0.92*	0.46	−0.06	2.46*	6.90*	6.71*
		Depression	0.97*	0.92	0.12	6.06*	2.43	3.41*
		Behavior	0.64	0.63	0.97	1.27	0.80	0.11
		Quality of Life	NA	1.26	0.20	NA	3.76*	5.64*
ROC Curve		AUC	0.83	0.71	0.70	0.87	0.79	0.77
		Balanced Accuracy	0.76	0.69	0.67	0.78	0.76	0.74
		Sensitivity	0.77	0.65	0.74	0.80	0.81	0.67
		Specificity	0.75	0.72	0.60	0.77	0.71	0.81

Notes: V1=Visit 1 (∼7 days post-injury); V2=Visit 2 (∼4-months post-injury); V3=Visit 3 (∼1-year post-injury); Diagnostic (DX); ROC=Receiver operating characteristic; AUC=overall performance.

⁎ denotes features that were selected in the individual domain models.

**Table 4:** Variable Importance score (VIMP %) for the diagnostic and outcome models for the neurosensory domain: Symptom provocation and Performance-based measures.

Neurosensory Domain							
Metric	Tests	DX			Outcome		
		V1	V2	V3	V1	V2	V3
Symptom Provocation: Feature selection	Double Dorsal Foot Stretch	4.31*	1.61	−0.19	3.93*	3.81	6.24
	Smooth Pursuit	0.70	−0.27	−1.34	4.05*	19.48*	29.82*
	Horizontal Saccades	2.27	−0.22	−0.97	3.36*	4.52	6.43*
	Vertical Saccades	5.95*	0.70	−1.48	7.96	9.95*	12.14
	Horizontal Vestibular-Ocular Reflex	2.96*	0.83	−1.33	9.03*	11.47*	1.53
	Vertical Vestibular-Ocular Reflex	4.29*	0.83	−0.65	7.31*	22.18*	5.40
	Visual Motion Sensitivity	3.51*	1.02	−1.07	7.53*	13.24*	12.40
	Near Point Convergence	3.93*	0.41	−1.17	2.09	6.95*	11.16
	Monocular Accommodation	4.28*	−0.48	−1.30	3.27	2.77	14.79*
	Tandem Gait	5.20*	1.62	−0.77	6.74*	24.34*	5.11*
	King-Devick	4.60*	1.05	−0.63	7.56*	6.84	7.54*
ROC Curve	AUC	0.61	0.48	0.39	0.73	0.61	0.62
	Balanced Accuracy	0.65	0.60	0.56	0.71	0.66	0.69
	Sensitivity	0.65	0.45	0.37	0.74	0.60	0.64
	Specificity	0.64	0.75	0.74	0.69	0.71	0.74
Performance-based: Feature selection	Near Point Convergence	−0.26	0.78	−1.49	4.53	1.94	1.91
	Monocular Accommodation	1.09	0.56	−1.12	7.47*	1.21	7.25
	King-Devick _RT_	3.66*	2.30	1.93	0.87	1.70	−0.31
	King-Devick _Er_	1.14	−0.16	−0.59	3.94*	4.04	2.31
	Tandem Gait Forwards Open _Er_	0.20	0.08	0.06	1.51	0.04	0.55
	Tandem Gait Forwards Closed _Er_	0.81	−0.22	−1.48	−0.88	2.62	7.66*
	Tandem Gait Backwards Open _Er_	0.80	0.40	−0.66	1.87	−1.24	1.60
	Tandem Gait Backwards Closed _Er_	1.74	1.77	−1.08	0.19	−0.89	0.28
ROC Curve	AUC	0.61	0.54	0.46	0.65	0.45	0.51
	Balanced Accuracy	0.60	0.54	0.51	0.63	0.51	0.53
	Sensitivity	0.60	0.53	0.36	0.52	0.63	0.60
	Specificity	0.60	0.56	0.66	0.74	0.39	0.47

Notes: V1=Visit 1 (∼7 days post-injury); V2=Visit 2 (∼4-months post-injury); V3=Visit 3 (∼1-year post-injury); Diagnostic (DX); RT=Reaction Time; Er=Errors; ROC=Receiver operating characteristic; AUC=overall performance.

⁎ denotes features that were selected in the individual domain models.

**Table 5:** Variable Importance score (VIMP %) for the diagnostic and outcome models for the cognitive domain: Paper-and-pencil, and Computerized (Cogstate) tasks.

Cognitive Domain							
Metric	Tests	DX			Outcome		
		V1	V2	V3	V1	V2	V3
Paper-and-Pencil tasks: Feature Selection	HVLT-R Immediate Recall	2.84*	3.98*	1.93	−0.45	−0.34	0.74
	HVLT-R Delayed Recall	2.48*	2.74*	1.87	1.38	1.04	−0.07
	Letter Fluency	1.19	0.05	0.57	−0.51	0.63	0.19
	Stroop Color Naming	1.35	0.45	−0.10	0.13	0.64	2.05
	Stroop Word Reading	0.44	0.57	0.30	0.10	1.90	1.33
	Stroop Inhibition	2.43*	1.31	0.38	−0.18	0.22	3.19*
	Stroop Inhibition/Switching	0.33	0.48	0.23	0.65	1.01	0.79
	Trails A	0.59	−0.34	0.62	1.44	1.91	1.41
	Trails B	1.02	0.92	0.33	0.09	3.08	0.88
	Trails B _Er_	0.42	0.18	0.32	0.17	0.71	0.43
	Digit Span	1.72	1.41	0.54	0.61	2.98	2.12
	Coding	0.98	0.25	0.08	−0.05	0.26	2.79*
	Symbol Search	1.48	−0.01	−0.03	−0.02	1.23	0.58
ROC Curve	AUC	0.69	0.64	0.61	0.50	0.56	0.46
	Balanced Accuracy	0.66	0.61	0.58	0.52	0.56	0.50
	Sensitivity	0.69	0.62	0.58	0.61	0.55	0.57
	Specificity	0.62	0.59	0.59	0.42	0.58	0.43
Cogstate: Feature Selection	Identification _RT_	0.64	1.59	−0.20	2.00	2.86	4.34*
	Identification _ac_	−0.62	−0.10	0.75	1.09	2.11	0.90
	Detection _RT_	0.26	1.10	0.09	−0.15	0.54	3.10
	Detection _ac_	−0.26	0.18	0.00	0.49	0.64	3.75*
	One-Card Learning _RT_	−0.59	0.01	−0.85	0.17	2.19	5.00*
	One-Card Learning _ac_	1.09	1.34	1.84	−1.08	2.69	0.39
	One-Back _RT_	0.11	−0.23	0.75	0.10	3.20	1.83
	One-Back _ac_	1.03	0.74	−0.05	0.56	1.52	−0.32
ROC Curve	AUC	0.56	0.57	0.54	0.54	0.47	0.60
	Balanced Accuracy	0.57	0.57	0.54	0.55	0.53	0.59
	Sensitivity	0.48	0.56	0.60	0.52	0.68	0.50
	Specificity	0.66	0.57	0.49	0.57	0.38	0.68

Notes: V1=Visit 1 (∼7 days post-injury); V2=Visit 2 (∼4-months post-injury); V3=Visit 3 (∼1-year post-injury); Diagnostic (DX); HVLT-*R*=Hopkins Verbal Learning Test Revised; Stroop=Delis-Kaplan Executive Function System (DKEFS) Color-Word Interference; Trails *A*=DKEFS Trail Making Number Sequence; Trails *B*= DKEFS Trail Making Number Letter Sequence; ER=Errors; RT=Reaction Time; ac=Accuracy; ROC=Receiver operating characteristic; AUC=overall performance.

⁎ denotes features that were selected in the individual domain models.

**Fig. 2:**
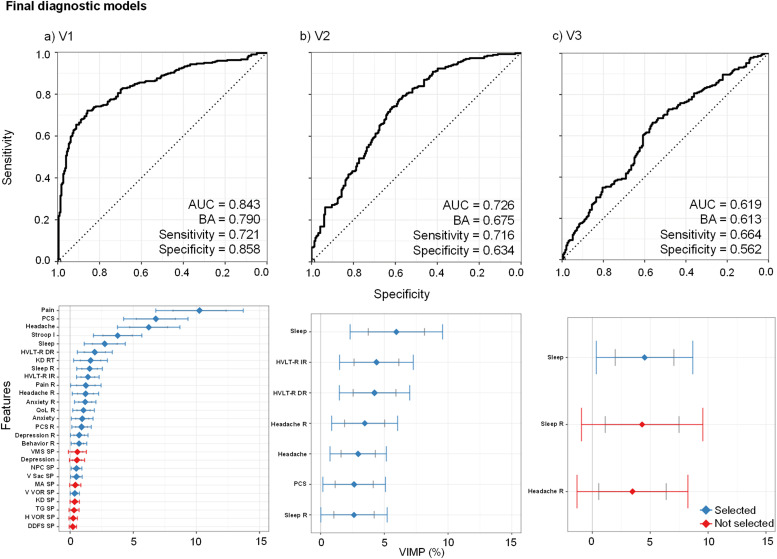
Results from the Random Forest analyses for the final diagnostic models at each Visit (V) for classifying pediatric “mild” traumatic brain injury patients versus healthy controls. The top row shows receiver operating characteristics (ROC) results including area under the curve (AUC), balanced accuracy (BA), sensitivity, and specificity for each visit. The bottom row displays the variable importance (VIMP) score for each variable in the final models at each visit. Feature selection for the final model occurred based on whether the lower bound of the 90 % VIMP confidence interval was greater than 0 % (selected = blue; not selected = red). For Visit 1, current/retrospective clinical-ratings predominated for feature selection along with memory and executive function on paper-and-pencil cognitive tests. In contrast, symptom provocation ratings from multiple neurosensory tests were eliminated in the final model. Somatic complaints (sleep and headache), overall post-concussive symptom burden, and performance on memory tests exhibited the best diagnostic accuracy at visits 2 and 3. The following abbreviations are included in the figure: Post-Concussive Symptoms (PCS), Quality of Life (QoL), Retrospective (R), Immediate (IR) and Delayed Recall (DR) from the Hopkins Verbal Learning Test Revised (HVLT-R), Stroop Inhibition (Stroop I), Symptom Provocation (SP), King-Devick (KD), Reaction Time (RT), Visual Motion Sensitivity (VMS), Near Point Convergence (NPC), Vertical Saccades (V Sac), Monocular Accommodative amplitude (MA), Vertical Vestibular Ocular Reflex (V VOR), Tandem Gait (TG), Horizontal Vestibular Ocular Reflex (H VOR), Double Dorsal Foot Stretch (DDFS).

The final V1 diagnostic model (see Fig. 2a) achieved good classification accuracy (0.84), with higher specificity (0.86) relative to sensitivity (0.72). Current self-reported clinical-ratings (except depression) and all self-reported retrospective-ratings, and performance on tests of immediate and delayed recall (HVLT-R), inhibition (Stroop), and rapid number naming (King-Devick), were retained for the final model. Somatic symptoms (pain, headache, PCS, sleep) were among the best predictors. In contrast, most symptom provocation measures from the neurosensory battery were excluded, with the exception of near-point convergence, vertical saccades, and vertical vestibular ocular reflex, as a result of lower VIMP scores.

#### Visit 2 (Approximately 4-months post-injury)

Random Forest analyses for diagnostic classification at V2 indicated that somatic features from the self-reported clinical-ratings domain (i.e., current and retrospective ratings of sleep and headache) and current ratings on the PCS scale were identified as important features, yielding good classification accuracy (see Table 3). HVLT-R immediate and delayed recall were also retained based on the VIMP criterion for the paper-and-pencil cognitive tests, resulting in a moderate classification accuracy for this domain (see Table 5).

The final V2 model demonstrated good overall classification, with an AUC of 0.73 and similar sensitivity (0.72) and reduced specificity (0.63) relative to V1 results. All features from individual domains that met the VIMP feature selection criterion were retained in the final model (see Fig. 2b).

#### Visit 3 (Approximately 1-Year post-injury)

At V3, only self-reported current and retrospective ratings of sleep, along with retrospective ratings of headache, were identified as important features from the clinical-ratings domain, yielding a moderate AUC (see Table 3). No features from any of the other domains exceeded the minimum VIMP criterion.

The final V3 model indicated a moderate overall classification AUC of 0.62, with sensitivity and specificity of 0.66 and 0.56, respectively. Only current ratings of sleep met the feature selection criteria, with the lower bound of the VIMP confidence interval dropping below 0 for retrospective measures of sleep and headache (see Fig. 2c).

### Outcome classification

#### Visit 1 (Approximately 1-week post-injury)

Random Forest analyses for the outcome model (favorable vs. poor outcome based on PSCI scores) at V1 resulted in nearly all features from the clinical-ratings domain (exceptions included retrospective and current measures of behavior, and retrospective measures of pain and anxiety) and neurosensory symptom provocation measures (exceptions included vertical saccades, near point convergence, and monocular accommodative amplitude) being selected based on the VIMP criterion, with both domains exhibiting good outcome classification accuracy. Somatic symptoms again predominated, although emotional disturbance (i.e., depression and anxiety) demonstrated higher classification accuracy for determining outcome status relative to diagnostic status. For performance-based measures from the neurosensory battery, outcome classification accuracy was moderate, with only King-Devick (error) and monocular accommodative amplitude features meeting the VIMP selection criterion. No features were selected from either the paper-and-pencil or computerized cognitive domains for V1. Finally, the injury characteristics model identified both the 5P risk score and number of previous injuries as important features, with a good AUC (see Table S1).

**Fig. 3:**
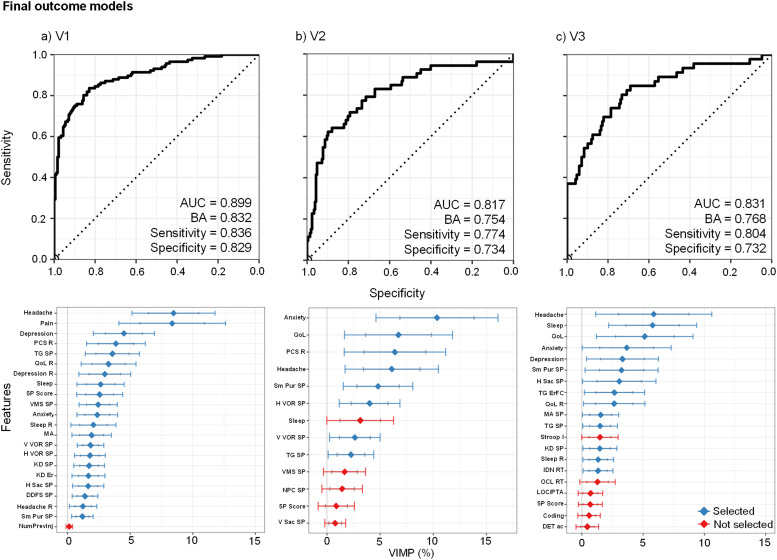
Results from the Random Forest analyses for the final outcome models at each Visit (V) for classifying pediatric “mild” traumatic brain injury patients with poor versus favorable outcomes. The top row shows receiver operating characteristics (ROC) results including area under the curve (AUC), balanced accuracy (BA), sensitivity, and specificity for each visit. The bottom row displays the variable importance (VIMP) score for each variable in the final models. Feature selection for the final model occurred based on whether the lower bound of the 90 % VIMP confidence interval was greater than 0 % (selected = blue; not selected = red). Across all three visits, current clinical-ratings, in particular somatic complaints (headache and sleep) and emotional distress, together with neurosensory symptom provocation measures, predominated for feature selection. In contrast, injury severity characteristics and performance-based cognitive measures tended to be eliminated in the final models. The following abbreviations are included in the figure: Post-Concussive Symptoms (PCS), Quality of Life (QoL), Retrospective (R), Symptom Provocation (SP), Tandem Gait (TG), Visual Motion Sensitivity (VMS), Monocular Accommodative amplitude (MA), Horizontal and Vertical Vestibular Ocular Reflex (H VOR; V VOR), King-Devick (KD), Error (Er), Horizontal and Vertical Saccades (H Sac; V Sac), Double Dorsal Foot Stretch (DDFS), Smooth Pursuit (Sm Pur), Near Point Convergence (NPC), Number of Previous Injuries (NumPrevInj), Stroop Inhibition (Stroop I), Identification (IDN), One-card Learning (OCL), Detection (DET), Reaction Time (RT), Accuracy (ac), Loss of Consciousness/Posttraumatic Amnesia (LOC/PTA).

The final model selected all features, except for number of previous injuries (see Fig. 3a), achieving an AUC of 0.90, with similar rates of sensitivity (0.84) and specificity (0.83).

#### Visit 2 (Approximately 4-months post-injury)

Random Forest analyses at V2 indicated a good AUC for the clinical-ratings domain, with anxiety, headache, quality of life, sleep and retrospective PCS ratings meeting the VIMP selection criterion (see Table 3). Several symptom provocation measures in the neurosensory domain were also selected, and yielded a moderate AUC (see Table 4). Similar to V1, no features were selected from paper-and-pencil or computerized cognitive testing, which yielded low AUCs. Only the 5P risk score was identified as an important feature from the injury characteristics model, which exhibited low classification accuracy (see Table S1).

The final V2 outcome classification model demonstrated a good AUC (0.82), and similar levels of sensitivity (0.77) and specificity (0.73). All clinical measures, except for current ratings of sleep, met the VIMP selection criterion and were among the best predictors. In contrast, three of the seven neurosensory symptom provocation measures and the 5P risk score did not meet criteria for selection in the final V2 outcome model (see Fig. 3b).

#### Visit 3 (Approximately 1-Year post-injury)

Random Forest analyses for outcome classification at V3 selected several retrospective and current clinical measures (somatic and emotional), with the clinical model showing good overall discrimination (see Table 3). The neurosensory symptom provocation model demonstrated moderate discrimination (see Table 4) with fewer features selected relative to earlier visits. The overall AUC for performance-based neurosensory measures was low, with only tandem gait errors (forward eyes closed) meeting the VIMP criterion for feature selection (see Table 4). Low classification accuracy was also observed for the paper-and-pencil cognitive tests, with Stroop inhibition and coding selected as features based on the VIMP criterion. In contrast to V1 and V2, the computerized testing model for outcome classification demonstrated a moderate AUC (see Table 5), with reaction times on one-card learning and identification tasks, and accuracy on the detection task, meeting selection criteria. Finally, both the 5P risk score and LOC/PTA were selected from the injury characteristics model in spite of a low AUC (see Table S1).

The final V3 model demonstrated a good classification AUC of 0.83 (see Fig. 3c), with higher sensitivity (0.80) relative to specificity (0.73). All current/retrospective clinical features, neurosensory symptom provocation (i.e., smooth pursuit, horizontal saccades, monocular accommodative amplitude, tandem gait, and King-Devick), and performance-based measures (tandem gait errors forward eyes closed) were selected in the final model based on the VIMP criterion. None of the computerized or paper-and-pencil cognitive tests were identified as important features in the final model with the exception of reaction time on the identification test, and the injury characteristics measures were similarly eliminated.

## Discussion

Clinical evaluations spanning multiple cognitive, behavioral, and neurosensory domains are commonly used to diagnose acute concussion, as well as predict poor versus favorable outcomes months post-injury. However, the optimal tests, or combination of tests, that are the most effective for diagnosing pmTBI and determining outcomes, and how their utility evolves over time post-injury, remains unclear (Polinder et al., 2018). Moreover, tests within each domain and across domains are frequently collinear, which may decrease their clinical utility and squander valuable assessment time. Unlike prior studies that primarily focused on group differences in mean performance (Kooper et al., 2024; McConnell et al., 2020), the current study used machine learning to determine the clinical assessments that were most effective at individual-level classification accuracy. Results suggested that overall diagnostic classification accuracy was highest at one-week post-injury (∼84 %), and then steadily declined as a function of time post-injury up through 1-year (∼62 %), most likely as a result of natural recovery across the assessed domains (Ledoux et al., 2019). In contrast, although the percentage of pmTBI with poor outcomes decreased after the first visit (38.9 % at 7 days, 20.2 % at 4 months and 21.4 % at 1-year post-injury), the classification accuracy for the outcome models remained above 80 % for all post-injury visits. Current somatic symptom burden, and retrospective report of somatic and emotional disturbances, exhibited the highest predictive validity across all visits in both diagnostic and outcome models.

### Diagnostic classification accuracy

The diagnostic classification models indicated that metrics of subjective experience (i.e., self-report), including symptom provocation during neurosensory exams, outperformed performance-based measures across all visits. Specifically, at one-week post-injury, all self-reported retrospective and most concurrent clinical-ratings significantly contributed to classification accuracy as defined by our VIMP criterion within their individual domains. While a moderate degree of collinearity existed between self-report measures within clinical tests, strong positive correlations were observed between retrospective and current ratings of anxiety, depression, and behavior, indicating that post-injury symptoms may, in part, reflect pre-existing conditions. Specifically, retrospective assessments might partially reflect pre-existing personality traits, potentially providing insights into stable emotional and behavioral tendencies post-injury (Durish et al., 2018; Emery et al., 2016; Rosenbaum et al., 2020; Vasa et al., 2002).

High collinearity among symptom provocation measures during neurosensory testing suggests that these variables may be capturing a singular, non-specific construct, rather than distinct aspects of post-injury functioning. This included the double-dorsal foot stretch test, which was previously implemented as a control measure for non-neurosensory symptom provocation (Mayer, Wertz et al., 2020), but ultimately met the inclusion criteria within the neurosensory diagnostic model at V1. Thus, it is also not surprising that the sensory provocation measures did not perform well in the final diagnostic model for sub-acute injuries when all features from individual domains were considered collectively, whereas all of the clinical-rating scales were retained.

Both concurrent and retrospective self-reported clinical-ratings of somatic symptoms, particularly headache and sleep disturbances, emerged as the best features for predicting diagnostic classification accuracy at 4 months, with concurrent sleep issues selected as the only relevant feature in the diagnostic model at 1-year post-injury. Post-traumatic headache is a common complaint following pediatric TBI regardless of injury severity (Durish et al., 2018; McConnell et al., 2020), and is often accompanied by heightened reports of sleep, mood, sensory, and cognitive disturbances (Durish et al., 2018; McConnell et al., 2020). Moreover, a recent meta-analysis found that sleep disturbances in pmTBI remain elevated for several months post-injury (Djukic et al., 2022). Current findings extend these results for up to a year post-injury, highlighting the prolonged impact of post-injury sleep disturbances and underscoring the need for further research on how both retrospective and concurrent sleep ratings contribute to pmTBI.

Performance-based measures showed less evidence of collinearity across both cognitive (paper-and-pencil tests and computerized versions) and neurosensory measures and thus likely measured independent constructs. However, performance-based measures generally had much lower diagnostic sensitivity and specificity. The main exception was long-term memory retrieval, which improved diagnostic classification accuracy at both V1 and V2, whereas executive dysfunction contributed only at V1. Previous studies suggest that “mild” TBI in young adults can result in persistent memory deficits and increased psychological strain during cognitive tasks, months or years post-injury (Cox & Fernandes, 2024; Konrad et al., 2011; Kooper et al., 2024). Similarly, recent findings indicate that higher self-reported concussion symptoms were associated with poorer verbal recall and recognition memory in adolescents (Jones et al., 2023). Relatedly, sleep disturbance during early neurodevelopment has been linked to impaired memory consolidation (Tononi & Cirelli, 2014), which may help explain the ongoing challenges with memory and concentration reported by individuals with pmTBI in everyday activities (Ponsford et al., 2011; Sumpter et al., 2013; Williams et al., 2022). In contrast to previous findings suggesting improved classification accuracy relative to paper-and-pencil tests (Chadwick et al., 2021; Sicard et al., 2022), computerized cognitive testing did not contribute to diagnostic classification at any of the visits in the current study. The only exception was the King-Devick test, a computerized measure of rapid number naming, which demonstrated diagnostic utility at V1. This aligns with previous research indicating that the King-Devick test is most sensitive during the acute phase of injury (Silverberg et al., 2014).

### Outcome classification accuracy

Many of the same patterns observed in diagnostic classification were present for the outcome models, although key differences also emerged. Foremost, evaluations of subjective experience again outperformed performance-based measures across all visits in terms of classification accuracy. This is not surprising, as poor versus favorable outcome was determined by self-reported PCS as is routinely done in clinical practice (Chadwick et al., 2022), and moderate correlation existed among the measures. Unlike diagnostic classification, cognitive functioning was not predictive of outcome at one-week post-injury, suggesting that these domains are rapidly decoupled (Sicard et al., 2024).

Similar to diagnostic classification models, concurrent ratings of somatic symptoms remained influential for the classification of outcome status for all visits. In contrast, emotional distress, specifically self-reported depression and anxiety, emerged as key features for determining poor outcomes post-pmTBI. Moreover, unlike diagnostic classification models, where clinical scales of somatic and emotional distress contributed only at V1, these emotional measures continued to predict outcomes at V2 and V3. This aligns with previous studies linking emotional vulnerability to prolonged recovery and the exacerbation of other concussion symptoms (Chendrasekhar, 2019; Djukic et al., 2022; Rosenbaum et al., 2020). Moreover, this prolonged influence also affected overall QOL, which was lower in patients with poor outcomes, consistent with previous evidence (Novak et al., 2016; Tham et al., 2013). Additionally, symptom provocation was also a better predictor at later visits both within its own domain and in final outcome models (less drop out) relative to diagnostic models. Previous studies have found abnormal VOMS, in addition to history of anxiety or depression and longer days post-injury, as predictors of higher emotional loads post pediatric concussion (Johnson et al., 2024). Thus, patients with poor outcomes remain more sensitive to tests of provocation over extended periods of recovery (Alkathiry et al., 2019; Crampton et al., 2022; Mayer, Wertz et al., 2020), which has great relevance for its routine use in tests of exercise as a metric of recovery (Leddy et al., 2019).

For performance-based measures, errors on the King-Devick test and monocular accommodative amplitude contributed to outcome classification at V1. Cognitive (reaction time on the computerized identification task) and neurosensory (errors on tandem gait in the forward eyes-closed condition) performance-based measures significantly contributed to outcome classification at V3, with no evidence of improving accuracy at V2. These findings align with previous studies identifying tandem gait as a predictor of recovery duration (Howell et al., 2018; Mayer, Wertz et al., 2020; Zemek et al., 2016), as well as studies indicating that attentional impairments may be more pronounced in children with poorer outcomes (Babikian et al., 2011; Robertson-Benta et al., 2023). Among injury severity characteristics, the 5P risk score was the only measure selected at V1. The 5P risk score was developed to stratify the risk of persisting PCS in children and youth using readily available clinical features, with early post-concussion 5P risk score serving as a predictor of symptoms lasting beyond one month (Howell et al., 2018; Zemek et al., 2016). Current findings extend these results in terms of being associated with poor versus favorable outcomes in the early stages of pmTBI.

### Limitations

Strengths of the study include a diverse, large sample that was prospectively assessed at three homogeneous post-injury intervals, as well as repeated assessments in a diverse, large age- and sex-matched healthy control cohort to control for repeat assessment effects. Limitations include reliance on a single computerized battery to measure cognitive functioning, which precludes direct comparisons with other available tools. While previous studies in early phases of injury with adults have generally found no one computerized battery to outperform others (Czerniak et al., 2020; Nelson et al., 2017), the use of a broader range of computerized batteries could provide more robust insights on their putative benefits for diagnostic and outcome prediction in children. Second, the neurosensory battery did not assess auditory functioning, which could capture additional facets of pmTBI pathology, such as auditory processing deficits (e.g., tinnitus) or impairments affecting multiple sensory modalities, commonly seen in mTBI populations (Lew et al., 2010; Raza et al., 2024). Third, our sample did not include a comparison group of children with orthopedic injuries, a group that can better account for nonspecific injury-related symptoms and premorbid risk factors. For example, prior studies using orthopedically injured controls reported rates of PSaC that are higher than those observed in HC but still lower than pmTBI (Ewing-Cobbs et al., 2018; Mayer et al., 2023; Yeates et al., 2012). Lastly, test administrators were not blinded to participants’ diagnoses, which may have introduced bias into the assessments.

### Conclusion

The current study highlights the need for a multidimensional, time-sensitive approach to clinical assessments following pmTBI, both in terms of diagnostic and outcome accuracy. Clinicians should prioritize a combination of clinical ratings, especially of headache severity, sleep, and emotional disturbances, with cognitive tests focused on memory and executive functioning, as well as neurosensory tests such as the King-Devick, tailored to specific post-injury intervals in both diagnostic and outcome assessments up to 4 months post injury. Retrospective symptom assessments, likely to be indicative of trait-like phenomenon, emerged as being valuable for identifying individuals at risk of poor outcomes even up to 1-year post-injury, enabling earlier and more targeted interventions. Computerized cognitive testing, and most neurosensory measures, showed minimal diagnostic or outcome prediction after 1-week post-injury with the exception of the King Devick and tandem gait. Ultimately, these results imply a need for strategically selected tests at different stages post-injury to enhance clinical care, shorten assessment batteries, and understand recovery trajectories for children and adolescents with “mild” TBI. Future studies should consider incorporating a broader range of objective measures, including neuroimaging and blood-based biomarkers, to provide a more comprehensive evaluation of injury and recovery trajectories.

## Declaration of competing interest

The authors declare the following financial interests/personal relationships which may be considered as potential competing interests:

Andrew R. Mayer reports financial support was provided by National Institutes of Health. If there are other authors, they declare that they have no known competing financial interests or personal relationships that could have appeared to influence the work reported in this paper.

## Appendix Supplementary materials

Image, application 1
