# Supplementary material for: Optimizing pediatric “Mild” traumatic brain injury assessments: A multi-domain random forest analysis of diagnosis and outcomes
Source: Int J Clin Health Psychol. 2025 Jun 27;25(3):100600. doi: 10.1016/j.ijchp.2025.100600 (PMC12269848; doi:10.1016/j.ijchp.2025.100600)
Supplement: Supplementary file 1 [file mmc1.docx]

Supplemental materials for:

**Optimizing Pediatric “Mild” Traumatic Brain Injury Assessments: Multi-Domain Random Forest Analysis of Diagnosis and Outcomes**

**Methods**

***Participants***

A total of 338 patients with pediatric “mild” traumatic injury (pmTBI) and 257 healthy children (HC) were recruited and included at V1. Exclusionary criteria included medical condition/positive radiology findings, positive drug screen (including amphetamines, methamphetamines, benzodiazepines, barbiturates, cocaine, marijuana, methadone, opiates, phencyclidine, and MDMA), and/or psychiatric/neurological disorders. At V1, medical condition/positive radiology findings excluded 3 participants (1 pmTBI; 2 HC), a positive drug screen excluded 4 participants (2 pmTBI; 2 HC), and psychiatric/neurological disorders excluded 5 participants (4 pmTBI; 1 HC). Moreover, 14 participants withdrew (6 pmTBI; 8 HC) and 2 pmTBI participants were excluded due to communication issues. This resulted in a final clinical sample of 323 pmTBI and 244 HC at V1.

Attrition occurred for 52 pmTBI (84% retention) and 14 HC (94.3% retention) between V1 and V2. Due to COVID-19 pandemic restrictions, 1 pmTBI was unable to complete the study. This resulted in 270 pmTBI and 230 HC eligible to participate at V2. At V2, 5 participants were excluded due to communication issues (3 HC) and psychiatric/neurological disorder (2 HC). In addition, 4 participants were excluded for a positive drug screen (1 pmTBI; 2 HC), and 5 pmTBI were unable to follow up for V2 only and returned for V3. This resulted in 264 pmTBI and 223 HC eligible to participate at V2.

From this subset, attrition occurred for 28 pmTBI (89.6% retention from V2) and 15 HC (93.3% retention from V2) between V2 and V3. Three pmTBI participants were further lost to follow-up due to COVID-19 pandemic restrictions. This resulted in 238 pmTBI and 208 HC eligible at V3. For eligible V3 participants, 11 were excluded, 4 due to a positive drug screen (1 pmTBI; 3 HC), 2 pmTBI due to a medical condition/positive radiology finding, and 5 HC due to psychiatric/neurological disorders which resulted in 235 pmTBI and 200 HC at V3. Although retention rates from V1 to V2 and from V2 to V3 were relatively high based on exclusion criteria, by V3 there was a notable decrease in sample size, with only 74.6% of recruited pmTBI and 85.2% of the recruited HC, being eligible for final analysis at V3.

***Common Data Element Measures***

Patients and HC were administered a full battery of clinical and cognitive measures at all three visits. Clinical measures included medical history, the New Mexico Assessment of Pediatric Traumatic Brain Injury semi-structured interview (Hergert et al., 2022), self-report of Tanner stage of development (Kriz et al., 2016), and the Glasgow Outcome Scale Extended (GOS-E) Pediatric Revision (Beers et al., 2012). GOS-E was not included in the current analysis, but was collected as a recommended Common Data Element. Additional measures encompassed self and parent reports of concussion symptom severity, behavior, and quality of life for retrospective and current periods, including Post-Concussive Symptom Inventory (PCSI; Gioia et al., 2008; Gioia et al., 2009), Conflict and Behavioral Questionnaire (CBQ; Prinz et al., 1979), and Pediatric Quality of Life Inventory (PedsQL - Generic Core; Varni et al., 1999). With the author’s permission, the PCSI was adapted to be administered to 12 year old participants using the 13-18 year-old version, and references to an injury were removed to counter reporting bias in HC. Additionally, the revised version specified retrospective (i.e., one month prior to initial visit) and V1 (day of initial visit) reporting instructions.

Participants also independently completed current and retrospective Patient Reported Outcomes Measurement Information System (PROMIS) measures for sleep (Buysse et al., 2010), anxiety, and depression (Pilkonis et al., 2011), a brief numerical pain scale rating (0-10 Likert scale; Farrar et al., 2001), and Headache Impact Test (HIT; Kosinski et al., 2003). Parents alone filled out the Strengths and Difficulties Questionnaire (SDQ; Goodman, 1997) for current and retrospective period and the Brief Symptom Inventory (BSI-18; Derogatis & Fitzpatrick, 2004) as a measure of parental distress. Cognitive measures included selected tests (see Table 1) from the Delis-Kaplan Executive Function System (DKEFS; Delis et al., 2001), the Hopkins Verbal Learning Test Revised (HVLT-R), and the Wechsler Intelligence Scales depending on initial age at assessment. Specifically, the Wechsler Adult Intelligence Scale-IV (WAIS-IV; Wechsler, 2008) was used for participants 16-18 years old at enrollment whereas the Wechsler Intelligence Scale for Children-V (WISC-V; Wechsler, 2014) was used for participants 8-15 years old at enrollment.

***Persisting Symptoms after Concussion (PSaC)***

All PCSI data were normalized to a percentage value (sum of individual rating/maximum score * 100) to account for differences in scale ratings for various age ranges (Sady et al., 2014). Consistent with our previous publication (Mayer et al., 2020), pmTBI with poor outcomes were identified using a standardized rather than simple change method due to superior psychometric properties. Specifically, normalized PCSI total scores were first transformed to z-scores using the mean and standard deviation from the HC sample based on data from V1 only. Individuals from pmTBI were then binarily categorized into highly symptomatic (poor outcome) or non-symptomatic (favorable outcome) groups based on a corrected z-score threshold of z>1.64 (Mayer et al., 2020), separately for each visit.

***5P risk score***

A modified version of the 5P risk score (Zemek et al., 2016) was calculated by taking into account the following predictors: age, sex, symptomatic prior concussion, diagnosed migraine history, answering questions slowly, tandem gait errors, headache, sensitivity to noise, and fatigue. In general, the scoring system coded 1 as the presence and 0 for absence of symptoms or history (i.e., for any prior concussion with symptoms lasting more than 7 days, migraine history, answering questions slowly, greater than 3 total tandem gait errors, headache, or sensitivity to noise). Variables that are associated with higher risk were coded distinctively (i.e., being female (2), reporting feeling fatigued following injury (2), or increased age (5-7 years old: 0; 8-12 years old: 1; 13-18 years old: 2). The total risk points were aggregated to generate an overall 5P risk score, which ranged from 1 to 12 in this study due to the specified age inclusion criteria (8-18 years old). The risk score for this study was modified based on availability of data by using a semi-structured interview with the PCSI as the symptom scale instead of the number/duration of prior concussions derived from the Acute Concussion Evaluation (Gioia et al., 2009) and replacing a Balance Error Scoring System with total tandem gait errors under an identical error threshold (i.e., greater than 3).

**Results**

***Diagnostic classification – parent ratings***

Results from the diagnostic model based on parent ratings at V1 achieved good classification accuracy (see Table S9) and identified post-concussion symptom (PCS) rating as the most important feature, with retrospective ratings of PCS and retrospective Quality of Life (QoL), and current behavior ratings, also meeting selection criteria. At V2, parent ratings of PCS, and both current and retrospective ratings of QoL, met selection criteria resulting in a moderate classification accuracy. At V3, only current PCS ratings were selected, yielding a moderate AUC (see Table S9).

***Outcome classification – parent ratings***

Results from the outcome model (favorable vs. poor outcome based on PCSI scores) based on parent ratings demonstrated moderate classification accuracy across all three visits (see Table S9). At V1, only current PCS rating met selection criteria. At V2, the model selected current PCS rating, parent symptoms, and current and retrospective ratings of psychological attributes as important features. At V3, current ratings of PCS and psychological attributes were identified as important features in the model.

Table S1. Variable Importance score (VIMP %) for the outcome models for injury characteristics measures.

| **Metric** | **Measure** | **Outcome** | | |
| --- | --- | --- | --- | --- |
|  |  | **V1** | **V2** | **V3** |
| Injury Severity | 5P score | 31.35* | 13.36* | 8.79* |
|  | Number of Previous Injuries | 6.92* | 1.25 | 3.61 |
|  | Sports/Recreation Related | 0.98 | -1.05 | 2.01 |
|  | LOC/PTA | 3.11 | -0.42 | 8.34* |
| ROC curve | AUC | 0.72 | 0.50 | 0.56 |
|  | Balanced Accuracy | 0.65 | 0.58 | 0.57 |
|  | Sensitivity | 0.73 | 0.66 | 0.72 |
|  | Specificity | 0.57 | 0.50 | 0.43 |

V1=Visit 1 (~7 days post-injury); V2=Visit 2 (~4-months post-injury); V3=Visit 3 (~1-year post-injury); LOC/PTA=Loss of Consciousness/Posttraumatic Amnesia; ROC=Receiver operating characteristic; AUC=overall performance; * denotes features that were selected in the model at each visit.

Table S2. Demographic variables for socio-economic status (SES) based on annual family income, and race.

|  |  | V1 pmTBI | V1 HC |
| --- | --- | --- | --- |
|  |  | (N=323) | (N=244) |
| SES | Low (<$49,999) | 43.96% | 25.00% |
|  | Middle ($50,000-$74,999) | 14.86% | 17.21% |
|  | High (>$75,000) | 34.99% | 50.00% |
|  | Refused/Unknown | 6.19% | 6.97% |
|  | Missing | 0.00% | 0.82% |
| Race | American Indian/Alaska Native | 10.22% | 2.05% |
|  | Asian | 0.93% | 1.23% |
|  | Black or African American | 4.64% | 1.64% |
|  | Native Hawaiian or Other Pacific Islander | 0.93% | 0.41% |
|  | White | 68.73% | 87.30% |
|  | More than one race | 7.43% | 4.51% |
|  | Unknown/Not Reported | 4.95% | 1.23% |
|  | Missing | 2.17% | 1.64% |

Table S3. Correlation matrices for the clinical-ratings domain: retrospective and current child ratings.

| **V1** |  | **PCS R** | **SLP R** | **PN R** | **HD R** | **AX R** | **DP R** | **BH R** | **QoL R** | **PCS** | **SLP** | **PN** | **HD** | **AX** | **DP** | **BH** | **QoL** |
| --- | --- | --- | --- | --- | --- | --- | --- | --- | --- | --- | --- | --- | --- | --- | --- | --- | --- |
|  | **PCS R** |  | 0.37 | 0.42 | 0.54 | 0.54 | 0.51 | 0.22 | -0.59 | 0.60 | 0.34 | 0.33 | 0.36 | 0.43 | 0.45 | 0.26 |  |
|  | **SLP R** | 0.50 |  | 0.29 | 0.44 | 0.39 | 0.37 | 0.22 | -0.48 | 0.29 | 0.55 | 0.22 | 0.24 | 0.25 | 0.26 | 0.23 |  |
|  | **PN R** | 0.42 | 0.37 |  | 0.46 | 0.37 | 0.33 | 0.09 | -0.45 | 0.22 | 0.23 | 0.23 | 0.25 | 0.23 | 0.27 | 0.12 |  |
|  | **HD R** | 0.45 | 0.40 | 0.36 |  | 0.48 | 0.43 | 0.22 | -0.61 | 0.38 | 0.37 | 0.32 | 0.58 | 0.35 | 0.37 | 0.22 |  |
|  | **AX R** | 0.62 | 0.50 | 0.32 | 0.42 |  | 0.65 | 0.23 | -0.68 | 0.36 | 0.34 | 0.21 | 0.29 | 0.76 | 0.55 | 0.24 |  |
|  | **DP R** | 0.57 | 0.51 | 0.24 | 0.34 | 0.67 |  | 0.35 | -0.64 | 0.48 | 0.33 | 0.28 | 0.34 | 0.61 | 0.76 | 0.35 |  |
|  | **BH R** | 0.31 | 0.39 | 0.20 | 0.25 | 0.43 | 0.46 |  | -0.31 | 0.22 | 0.24 | 0.15 | 0.15 | 0.25 | 0.35 | 0.88 |  |
|  | **QoL R** | -0.61 | -0.61 | -0.41 | -0.57 | -0.62 | -0.57 | -0.40 |  | -0.41 | -0.41 | -0.22 | -0.41 | -0.57 | -0.56 | -0.32 |  |
|  | **PCS** | 0.86 | 0.54 | 0.40 | 0.45 | 0.65 | 0.58 | 0.36 | -0.66 |  | 0.49 | 0.63 | 0.59 | 0.46 | 0.59 | 0.27 |  |
|  | **SLP** | 0.48 | 0.82 | 0.30 | 0.41 | 0.47 | 0.49 | 0.37 | -0.62 | 0.54 |  | 0.43 | 0.48 | 0.41 | 0.49 | 0.26 |  |
|  | **PN** | 0.35 | 0.33 | 0.64 | 0.42 | 0.27 | 0.22 | 0.23 | -0.40 | 0.44 | 0.28 |  | 0.51 | 0.27 | 0.37 | 0.18 |  |
|  | **HD** | 0.43 | 0.31 | 0.34 | 0.81 | 0.40 | 0.29 | 0.17 | -0.54 | 0.45 | 0.35 | 0.38 |  | 0.39 | 0.49 | 0.14 |  |
|  | **AX** | 0.56 | 0.44 | 0.31 | 0.37 | 0.88 | 0.62 | 0.47 | -0.61 | 0.66 | 0.44 | 0.29 | 0.41 |  | 0.72 | 0.29 |  |
|  | **DP** | 0.53 | 0.43 | 0.22 | 0.31 | 0.63 | 0.89 | 0.48 | -0.55 | 0.60 | 0.47 | 0.21 | 0.30 | 0.66 |  | 0.38 |  |
|  | **BH** | 0.28 | 0.44 | 0.20 | 0.26 | 0.41 | 0.42 | 0.90 | -0.40 | 0.34 | 0.38 | 0.23 | 0.16 | 0.45 | 0.44 |  |  |
|  | **QoL** |  | | | | | | | | | | | | | | |  |
| **V2** |  | **PCS R** | **SLP R** | **PN R** | **HD R** | **AX R** | **DP R** | **BH R** | **QoL R** | **PCS** | **SLP** | **PN** | **HD** | **AX** | **DP** | **BH** | **QoL** |
|  | **PCS R** |  | 0.38 | 0.43 | 0.52 | 0.53 | 0.50 | 0.23 | -0.60 | 0.52 | 0.39 | 0.31 | 0.36 | 0.33 | 0.40 | 0.25 | -0.43 |
|  | **SLP R** | 0.51 |  | 0.27 | 0.45 | 0.40 | 0.37 | 0.27 | -0.48 | 0.23 | 0.52 | 0.16 | 0.32 | 0.20 | 0.23 | 0.25 | -0.32 |
|  | **PN R** | 0.43 | 0.33 |  | 0.47 | 0.39 | 0.37 | 0.09 | -0.44 | 0.29 | 0.22 | 0.19 | 0.35 | 0.19 | 0.28 | 0.08 | -0.26 |
|  | **HD R** | 0.45 | 0.37 | 0.35 |  | 0.45 | 0.42 | 0.23 | -0.65 | 0.38 | 0.34 | 0.18 | 0.58 | 0.25 | 0.28 | 0.21 | -0.40 |
|  | **AX R** | 0.57 | 0.47 | 0.35 | 0.39 |  | 0.64 | 0.23 | -0.66 | 0.39 | 0.39 | 0.21 | 0.37 | 0.55 | 0.50 | 0.26 | -0.52 |
|  | **DP R** | 0.58 | 0.50 | 0.26 | 0.32 | 0.66 |  | 0.36 | -0.62 | 0.41 | 0.36 | 0.23 | 0.41 | 0.46 | 0.61 | 0.34 | -0.49 |
|  | **BH R** | 0.36 | 0.35 | 0.29 | 0.26 | 0.53 | 0.54 |  | -0.32 | 0.23 | 0.32 | 0.17 | 0.29 | 0.21 | 0.23 | 0.72 | -0.30 |
|  | **QoL R** | -0.58 | -0.55 | -0.47 | -0.56 | -0.60 | -0.57 | -0.48 |  | -0.42 | -0.44 | -0.34 | -0.52 | -0.45 | -0.50 | -0.30 | 0.68 |
|  | **PCS** | 0.53 | 0.42 | 0.24 | 0.35 | 0.41 | 0.46 | 0.46 | -0.45 |  | 0.48 | 0.48 | 0.54 | 0.48 | 0.48 | 0.21 | -0.52 |
|  | **SLP** | 0.36 | 0.66 | 0.24 | 0.36 | 0.32 | 0.36 | 0.37 | -0.39 | 0.52 |  | 0.37 | 0.47 | 0.41 | 0.43 | 0.32 | -0.54 |
|  | **PN** | 0.28 | 0.18 | 0.48 | 0.30 | 0.20 | 0.11 | 0.17 | -0.33 | 0.21 | 0.16 |  | 0.43 | 0.36 | 0.44 | 0.23 | -0.51 |
|  | **HD** | 0.15 | 0.32 | 0.24 | 0.61 | 0.21 | 0.19 | 0.27 | -0.34 | 0.36 | 0.43 | 0.36 |  | 0.40 | 0.44 | 0.25 | -0.58 |
|  | **AX** | 0.43 | 0.39 | 0.26 | 0.30 | 0.58 | 0.58 | 0.54 | -0.59 | 0.54 | 0.39 | 0.25 | 0.23 |  | 0.71 | 0.27 | -0.71 |
|  | **DP** | 0.37 | 0.41 | 0.24 | 0.23 | 0.46 | 0.66 | 0.55 | -0.53 | 0.49 | 0.39 | 0.24 | 0.27 | 0.75 |  | 0.34 | -0.72 |
|  | **BH** | 0.32 | 0.35 | 0.25 | 0.22 | 0.46 | 0.50 | 0.83 | -0.39 | 0.44 | 0.40 | 0.17 | 0.26 | 0.47 | 0.57 |  | -0.36 |
|  | **QoL** | -0.48 | -0.52 | -0.39 | -0.41 | -0.44 | -0.53 | -0.45 | 0.70 | -0.55 | -0.49 | -0.34 | -0.43 | -0.66 | -0.67 | -0.45 |  |
| **V3** |  | **PCS R** | **SLP R** | **PN R** | **HD R** | **AX R** | **DP R** | **BH R** | **QoL R** | **PCS** | **SLP** | **PN** | **HD** | **AX** | **DP** | **BH** | **QoL** |
|  | **PCS R** |  | 0.36 | 0.42 | 0.51 | 0.55 | 0.53 | 0.29 | -0.60 | 0.48 | 0.28 | 0.37 | 0.38 | 0.37 | 0.37 | 0.22 | -0.39 |
|  | **SLP R** | 0.46 |  | 0.25 | 0.37 | 0.38 | 0.32 | 0.24 | -0.42 | 0.30 | 0.48 | 0.21 | 0.26 | 0.28 | 0.20 | 0.19 | -0.26 |
|  | **PN R** | 0.46 | 0.31 |  | 0.46 | 0.36 | 0.35 | 0.09 | -0.43 | 0.22 | 0.13 | 0.21 | 0.27 | 0.17 | 0.14 | -0.04 | -0.15 |
|  | **HD R** | 0.46 | 0.34 | 0.37 |  | 0.47 | 0.41 | 0.23 | -0.62 | 0.38 | 0.35 | 0.29 | 0.58 | 0.31 | 0.34 | 0.25 | -0.42 |
|  | **AX R** | 0.55 | 0.39 | 0.33 | 0.35 |  | 0.66 | 0.31 | -0.69 | 0.34 | 0.34 | 0.19 | 0.38 | 0.50 | 0.38 | 0.18 | -0.44 |
|  | **DP R** | 0.53 | 0.46 | 0.25 | 0.31 | 0.64 |  | 0.38 | -0.62 | 0.30 | 0.29 | 0.12 | 0.32 | 0.45 | 0.50 | 0.20 | -0.45 |
|  | **BH R** | 0.32 | 0.32 | 0.31 | 0.20 | 0.48 | 0.51 |  | -0.36 | 0.25 | 0.29 | 0.15 | 0.20 | 0.28 | 0.24 | 0.48 | -0.28 |
|  | **QoL R** | -0.58 | -0.53 | -0.49 | -0.52 | -0.56 | -0.56 | -0.43 |  | -0.45 | -0.38 | -0.29 | -0.48 | -0.47 | -0.42 | -0.24 | 0.61 |
|  | **PCS** | 0.52 | 0.43 | 0.24 | 0.31 | 0.45 | 0.48 | 0.38 | -0.39 |  | 0.54 | 0.43 | 0.53 | 0.57 | 0.59 | 0.24 | -0.64 |
|  | **SLP** | 0.34 | 0.56 | 0.19 | 0.32 | 0.33 | 0.37 | 0.41 | -0.43 | 0.48 |  | 0.34 | 0.52 | 0.57 | 0.51 | 0.37 | -0.61 |
|  | **PN** | 0.43 | 0.20 | 0.35 | 0.32 | 0.21 | 0.16 | 0.18 | -0.27 | 0.41 | 0.27 |  | 0.48 | 0.35 | 0.34 | 0.21 | -0.43 |
|  | **HD** | 0.17 | 0.25 | 0.18 | 0.50 | 0.17 | 0.20 | 0.18 | -0.25 | 0.33 | 0.36 | 0.26 |  | 0.50 | 0.54 | 0.26 | -0.64 |
|  | **AX** | 0.33 | 0.32 | 0.23 | 0.26 | 0.55 | 0.49 | 0.45 | -0.42 | 0.61 | 0.45 | 0.21 | 0.25 |  | 0.74 | 0.25 | -0.75 |
|  | **DP** | 0.36 | 0.36 | 0.26 | 0.27 | 0.43 | 0.62 | 0.44 | -0.46 | 0.61 | 0.43 | 0.21 | 0.22 | 0.73 |  | 0.30 | -0.73 |
|  | **BH** | 0.27 | 0.28 | 0.07 | 0.19 | 0.35 | 0.44 | 0.67 | -0.30 | 0.42 | 0.37 | 0.14 | 0.17 | 0.44 | 0.45 |  | -0.29 |
|  | **QoL** | -0.39 | -0.38 | -0.24 | -0.29 | -0.34 | -0.47 | -0.36 | 0.57 | -0.59 | -0.56 | -0.33 | -0.42 | -0.58 | -0.62 | -0.35 |  |

R=Retrospective Self-Report; PCS=Post-Concussion Symptoms (PCS); SLP=Sleep; PN=Pain; HD=Headache; AX=Anxiety; DP=Depression; BH=Behavior; QoL= Quality of Life; V1=Visit 1; V2=Visit 2; V3=Visit 3; Dotted grid=pmTBI/upper diagonal; Solid grid=HC/lower diagonal. **Collinearity: white r<0.40; yellow r=0.40-0.69; red r>0.70.**

Table S4. Correlation matrices for the neurosensory domain: symptom provocation measures.

| **V1** |  | **DDFS** | **Sm Pur** | **H Sac** | **V Sac** | **H VOR** | **V VOR** | **VMS** | **NPC** | **MA** | **TG** | **KD** |
| --- | --- | --- | --- | --- | --- | --- | --- | --- | --- | --- | --- | --- |
|  | **DDFS** |  | 0.81 | 0.68 | 0.65 | 0.44 | 0.45 | 0.39 | 0.49 | 0.56 | 0.52 | 0.47 |
|  | **Sm Pur** | 0.57 |  | 0.87 | 0.83 | 0.57 | 0.55 | 0.46 | 0.64 | 0.69 | 0.66 | 0.63 |
|  | **H Sac** | 0.31 | 0.80 |  | 0.95 | 0.65 | 0.64 | 0.54 | 0.68 | 0.68 | 0.68 | 0.72 |
|  | **V Sac** | 0.20 | 0.67 | 0.94 |  | 0.62 | 0.61 | 0.52 | 0.71 | 0.71 | 0.69 | 0.73 |
|  | **H VOR** | 0.22 | 0.66 | 0.90 | 0.92 |  | 0.95 | 0.80 | 0.47 | 0.46 | 0.50 | 0.44 |
|  | **V VOR** | 0.13 | 0.63 | 0.87 | 0.91 | 0.92 |  | 0.85 | 0.48 | 0.48 | 0.52 | 0.45 |
|  | **VMS** | 0.21 | 0.68 | 0.87 | 0.85 | 0.91 | 0.88 |  | 0.56 | 0.55 | 0.51 | 0.41 |
|  | **NPC** | 0.50 | 0.64 | 0.64 | 0.54 | 0.61 | 0.51 | 0.71 |  | 0.90 | 0.63 | 0.64 |
|  | **MA** | 0.65 | 0.54 | 0.40 | 0.32 | 0.37 | 0.29 | 0.48 | 0.82 |  | 0.66 | 0.68 |
|  | **TG** | 0.46 | 0.75 | 0.75 | 0.67 | 0.69 | 0.61 | 0.76 | 0.84 | 0.76 |  | 0.68 |
|  | **KD** | 0.62 | 0.65 | 0.55 | 0.46 | 0.51 | 0.46 | 0.54 | 0.69 | 0.64 | 0.76 |  |
| **V2** |  | **DDFS** | **Sm Pur** | **H Sac** | **V Sac** | **H VOR** | **V VOR** | **VMS** | **NPC** | **MA** | **TG** | **KD** |
|  | **DDFS** |  | 0.71 | 0.33 | 0.25 | 0.39 | 0.30 | 0.19 | 0.36 | 0.51 | 0.28 | 0.18 |
|  | **Sm Pur** | 0.54 |  | 0.71 | 0.58 | 0.59 | 0.55 | 0.48 | 0.51 | 0.60 | 0.31 | 0.28 |
|  | **H Sac** | 0.56 | 0.81 |  | 0.87 | 0.73 | 0.71 | 0.67 | 0.59 | 0.47 | 0.46 | 0.41 |
|  | **V Sac** | 0.40 | 0.76 | 0.90 |  | 0.77 | 0.75 | 0.78 | 0.66 | 0.44 | 0.50 | 0.50 |
|  | **H VOR** | 0.35 | 0.80 | 0.69 | 0.79 |  | 0.90 | 0.75 | 0.77 | 0.57 | 0.61 | 0.54 |
|  | **V VOR** | 0.43 | 0.82 | 0.75 | 0.77 | 0.93 |  | 0.81 | 0.75 | 0.58 | 0.56 | 0.52 |
|  | **VMS** | 0.38 | 0.73 | 0.72 | 0.78 | 0.91 | 0.93 |  | 0.78 | 0.50 | 0.66 | 0.62 |
|  | **NPC** | 0.32 | 0.82 | 0.71 | 0.73 | 0.82 | 0.86 | 0.84 |  | 0.67 | 0.77 | 0.67 |
|  | **MA** | 0.29 | 0.78 | 0.67 | 0.69 | 0.77 | 0.80 | 0.78 | 0.95 |  | 0.53 | 0.41 |
|  | **TG** | 0.27 | 0.73 | 0.67 | 0.66 | 0.77 | 0.78 | 0.80 | 0.91 | 0.88 |  | 0.74 |
|  | **KD** | 0.20 | 0.63 | 0.57 | 0.57 | 0.63 | 0.67 | 0.67 | 0.85 | 0.88 | 0.90 |  |
| **V3** |  | **DDFS** | **Sm Pur** | **H Sac** | **V Sac** | **H VOR** | **V VOR** | **VMS** | **NPC** | **MA** | **TG** | **KD** |
|  | **DDFS** |  | 0.50 | 0.23 | 0.16 | 0.29 | 0.28 | 0.16 | 0.28 | 0.23 | 0.29 | 0.35 |
|  | **Sm Pur** | 0.46 |  | 0.81 | 0.78 | 0.79 | 0.73 | 0.67 | 0.65 | 0.48 | 0.64 | 0.39 |
|  | **H Sac** | 0.35 | 0.84 |  | 0.91 | 0.80 | 0.73 | 0.75 | 0.62 | 0.45 | 0.55 | 0.27 |
|  | **V Sac** | 0.26 | 0.71 | 0.89 |  | 0.84 | 0.81 | 0.81 | 0.67 | 0.55 | 0.66 | 0.36 |
|  | **H VOR** | 0.21 | 0.59 | 0.70 | 0.81 |  | 0.94 | 0.89 | 0.79 | 0.61 | 0.81 | 0.46 |
|  | **V VOR** | 0.20 | 0.51 | 0.64 | 0.74 | 0.94 |  | 0.84 | 0.80 | 0.68 | 0.82 | 0.47 |
|  | **VMS** | 0.08 | 0.41 | 0.57 | 0.71 | 0.87 | 0.91 |  | 0.79 | 0.64 | 0.78 | 0.48 |
|  | **NPC** | 0.19 | 0.54 | 0.60 | 0.63 | 0.81 | 0.81 | 0.79 |  | 0.80 | 0.87 | 0.63 |
|  | **MA** | 0.28 | 0.59 | 0.56 | 0.59 | 0.66 | 0.62 | 0.58 | 0.86 |  | 0.77 | 0.63 |
|  | **TG** | 0.07 | 0.52 | 0.66 | 0.74 | 0.79 | 0.81 | 0.78 | 0.83 | 0.77 |  | 0.72 |
|  | **KD** | 0.23 | 0.62 | 0.71 | 0.73 | 0.81 | 0.82 | 0.75 | 0.80 | 0.73 | 0.90 |  |

DDFS=Double Dorsal Foot Stretch; Sm Pur=Smooth Pursuit; H Sac=Horizontal Saccades; V Sac=Vertical Saccades; H VOR=Horizontal Vestibular-Ocular Reflex;V VOR= Vertical Vestibular Ocular Reflex; VMS=Visual Motion Sensitivity; NPC=Near Point Convergence; MA=Monocular Accommodation; TW=Tandem Gait; KD=King-Devick; V1=Visit 1; V2=Visit 2; V3=Visit 3; Dotted grid=pmTBI/upper diagonal; Solid grid=HC/lower diagonal. **Collinearity: white r<0.40; yellow r=0.40-0.69; red r>0.70.**

Table S5. Correlation matrices for the neurosensory domain: performance-based measures.

| **V1** |  | **NPC** | **MA** | **KD_RT_** | **KD_ER_** | **TGFO_ER_** | **TGFC_ER_** | **TGBO_ER_** | **TGBC_ER_** |
| --- | --- | --- | --- | --- | --- | --- | --- | --- | --- |
|  | **NPC** |  | 0.65 | 0.17 | 0.15 | 0.06 | 0.07 | -0.01 | 0.12 |
|  | **MA** | 0.58 |  | 0.19 | 0.08 | -0.04 | 0.02 | -0.03 | 0.12 |
|  | **KD_RT_** | 0.02 | -0.07 |  | 0.14 | 0.18 | 0.09 | 0.23 | 0.22 |
|  | **KD_ER_** | -0.11 | -0.11 | 0.25 |  | 0.16 | 0.07 | 0.21 | 0.18 |
|  | **TGFO_ER_** | 0.07 | 0.04 | 0.01 | -0.03 |  | 0.47 | 0.60 | 0.34 |
|  | **TGFC_ER_** | 0.07 | 0.12 | 0.03 | 0.04 | 0.23 |  | 0.43 | 0.44 |
|  | **TGBO_ER_** | 0.00 | 0.08 | 0.04 | 0.05 | 0.30 | 0.34 |  | 0.49 |
|  | **TGBC_ER_** | -0.03 | 0.09 | 0.03 | 0.06 | 0.23 | 0.38 | 0.51 |  |
| **V2** |  | **NPC** | **MA** | **KD_RT_** | **KD_ER_** | **TGFO_ER_** | **TGFC_ER_** | **TGBO_ER_** | **TGBC_ER_** |
|  | **NPC** |  | 0.69 | -0.02 | 0.18 | -0.06 | 0.00 | 0.05 | -0.05 |
|  | **MA** | 0.63 |  | 0.14 | 0.22 | -0.04 | 0.01 | 0.04 | -0.06 |
|  | **KD_RT_** | 0.03 | -0.14 |  | 0.26 | -0.01 | -0.03 | 0.08 | 0.21 |
|  | **KD_ER_** | 0.02 | 0.04 | 0.18 |  | 0.07 | 0.04 | -0.05 | 0.03 |
|  | **TGFO_ER_** | -0.05 | 0.07 | 0.01 | 0.02 |  | 0.35 | 0.43 | 0.23 |
|  | **TGFC_ER_** | -0.01 | 0.05 | -0.06 | 0.07 | 0.28 |  | 0.35 | 0.25 |
|  | **TGBO_ER_** | 0.21 | 0.24 | 0.13 | 0.05 | 0.22 | 0.16 |  | 0.38 |
|  | **TGBC_ER_** | 0.24 | 0.14 | 0.07 | 0.08 | 0.11 | 0.23 | 0.24 |  |
| **V3** |  | **NPC** | **MA** | **KD_RT_** | **KD_ER_** | **TGFO_ER_** | **TGFC_ER_** | **TGBO_ER_** | **TGBC_ER_** |
|  | **NPC** |  | 0.70 | -0.07 | -0.10 | -0.05 | 0.02 | -0.05 | -0.05 |
|  | **MA** | 0.54 |  | -0.06 | -0.12 | 0.06 | 0.14 | -0.03 | 0.04 |
|  | **KD_RT_** | 0.02 | 0.04 |  | 0.34 | 0.27 | 0.16 | 0.20 | 0.03 |
|  | **KD_ER_** | -0.05 | -0.04 | 0.17 |  | 0.01 | -0.05 | 0.22 | 0.14 |
|  | **TGFO_ER_** | 0.04 | 0.08 | 0.05 | -0.05 |  | 0.51 | 0.38 | 0.33 |
|  | **TGFC_ER_** | 0.04 | 0.04 | -0.09 | -0.04 | 0.40 |  | 0.27 | 0.41 |
|  | **TGBO_ER_** | 0.02 | 0.07 | 0.10 | 0.04 | 0.17 | 0.23 |  | 0.54 |
|  | **TGBC_ER_** | -0.04 | 0.02 | -0.03 | -0.05 | 0.07 | 0.33 | 0.33 |  |

NPC=Near Point Convergence; MA=Monocular Accommodative amplitude; KD=King-Devick; TWFO=Tandem Gait Forward Open; TWFC=Tandem Gait Forward Closed; TWBO=Tandem Gait Backward Open; TWBC=Tandem Gait Backward Closed; RT=Reaction Time; ER=Errors; V1=Visit 1; V2=Visit 2; V3=Visit 3; Dotted grid=pmTBI/upper diagonal; Solid grid=HC/lower diagonal. **Collinearity: white r<0.40; yellow r=0.40-0.69; red r>0.70.**

Table S6. Correlation matrices for the cognitive domain: paper-and-pencil tasks.

| **V1** |  | **HVLT-R IR** | **HVLT-R DR** | **L Fluency** | **Stroop C** | **Stoop R** | **Stroop I** | **Stroop IS** | **Trails A** | **Trails B** | **Trails B_Er_** | **DS** | **Coding** | **Sy Se** |
| --- | --- | --- | --- | --- | --- | --- | --- | --- | --- | --- | --- | --- | --- | --- |
|  | **HVLT-R IR** |  | 0.52 | 0.21 | 0.19 | 0.17 | 0.18 | 0.06 | 0.15 | 0.27 | 0.13 | 0.25 | 0.23 | 0.23 |
|  | **HVLT-R DR** | 0.75 |  | 0.25 | 0.14 | 0.13 | 0.14 | 0.15 | 0.14 | 0.19 | 0.01 | 0.24 | 0.24 | 0.24 |
|  | **L Fluency** | 0.24 | 0.14 |  | 0.28 | 0.31 | 0.26 | 0.29 | 0.13 | 0.32 | 0.06 | 0.30 | 0.22 | 0.20 |
|  | **Stroop C** | 0.05 | 0.02 | 0.28 |  | 0.69 | 0.67 | 0.58 | 0.35 | 0.41 | 0.17 | 0.35 | 0.50 | 0.41 |
|  | **Stroop R** | 0.07 | 0.04 | 0.32 | 0.66 |  | 0.59 | 0.61 | 0.26 | 0.32 | 0.08 | 0.25 | 0.43 | 0.33 |
|  | **Stroop I** | 0.11 | 0.12 | 0.35 | 0.64 | 0.46 |  | 0.61 | 0.25 | 0.38 | 0.18 | 0.27 | 0.45 | 0.40 |
|  | **Stroop IS** | 0.00 | -0.02 | 0.26 | 0.46 | 0.44 | 0.59 |  | 0.31 | 0.40 | 0.17 | 0.29 | 0.46 | 0.40 |
|  | **Trails A** | 0.25 | 0.20 | 0.14 | 0.29 | 0.31 | 0.37 | 0.13 |  | 0.45 | 0.07 | 0.25 | 0.37 | 0.39 |
|  | **Trails B** | 0.20 | 0.18 | 0.30 | 0.31 | 0.34 | 0.46 | 0.26 | 0.46 |  | 0.60 | 0.42 | 0.52 | 0.52 |
|  | **Trails B_Er_** | 0.00 | 0.00 | 0.10 | 0.02 | 0.07 | 0.15 | 0.07 | 0.08 | 0.53 |  | 0.29 | 0.26 | 0.27 |
|  | **DS** | 0.20 | 0.14 | 0.29 | 0.22 | 0.19 | 0.34 | 0.16 | 0.27 | 0.32 | 0.13 |  | 0.31 | 0.32 |
|  | **Coding** | 0.13 | 0.15 | 0.22 | 0.36 | 0.39 | 0.39 | 0.37 | 0.35 | 0.45 | 0.10 | 0.18 |  | 0.56 |
|  | **Sy Se** | 0.22 | 0.17 | 0.25 | 0.42 | 0.44 | 0.43 | 0.42 | 0.43 | 0.44 | 0.09 | 0.24 | 0.62 |  |
| **V2** |  | **HVLT-R IR** | **HVLT-R DR** | **L Fluency** | **Stroop C** | **Stroop R** | **Stroop I** | **Stroop IS** | **Trails A** | **Trails B** | **Trails B_Er_** | **DS** | **Coding** | **Sy Se** |
|  | **HVLT-R IR** |  | 0.68 | 0.18 | 0.14 | 0.16 | 0.13 | 0.14 | 0.12 | 0.30 | 0.25 | 0.26 | 0.22 | 0.24 |
|  | **HVLT-R DR** | 0.75 |  | 0.19 | 0.09 | 0.10 | 0.10 | 0.12 | 0.17 | 0.24 | 0.15 | 0.15 | 0.20 | 0.23 |
|  | **L Fluency** | 0.26 | 0.34 |  | 0.20 | 0.32 | 0.22 | 0.28 | 0.16 | 0.35 | 0.24 | 0.32 | 0.22 | 0.28 |
|  | **Stroop C** | 0.11 | 0.08 | 0.30 |  | 0.63 | 0.68 | 0.57 | 0.35 | 0.40 | 0.12 | 0.23 | 0.40 | 0.36 |
|  | **Stroop R** | -0.02 | -0.04 | 0.31 | 0.75 |  | 0.50 | 0.56 | 0.25 | 0.33 | 0.16 | 0.25 | 0.38 | 0.32 |
|  | **Stroop I** | 0.20 | 0.17 | 0.36 | 0.67 | 0.48 |  | 0.62 | 0.30 | 0.42 | 0.21 | 0.24 | 0.37 | 0.37 |
|  | **Stroop IS** | 0.08 | 0.06 | 0.26 | 0.55 | 0.49 | 0.64 |  | 0.28 | 0.39 | 0.23 | 0.27 | 0.47 | 0.39 |
|  | **Trails A** | 0.08 | 0.07 | 0.09 | 0.31 | 0.31 | 0.24 | 0.29 |  | 0.42 | 0.03 | 0.18 | 0.37 | 0.38 |
|  | **Trails B** | 0.16 | 0.15 | 0.27 | 0.46 | 0.35 | 0.52 | 0.43 | 0.39 |  | 0.57 | 0.37 | 0.39 | 0.50 |
|  | **Trails B_Er_** | -0.06 | -0.06 | 0.09 | -0.01 | 0.00 | 0.04 | -0.01 | -0.09 | 0.37 |  | 0.24 | 0.20 | 0.20 |
|  | **DS** | 0.33 | 0.34 | 0.26 | 0.21 | 0.13 | 0.30 | 0.16 | 0.03 | 0.31 | 0.12 |  | 0.20 | 0.23 |
|  | **Coding** | 0.17 | 0.14 | 0.21 | 0.46 | 0.39 | 0.53 | 0.54 | 0.33 | 0.47 | 0.01 | 0.24 |  | 0.53 |
|  | **Sy Se** | 0.25 | 0.14 | 0.26 | 0.44 | 0.38 | 0.50 | 0.42 | 0.36 | 0.53 | 0.06 | 0.25 | 0.62 |  |
| **V3** |  | **HVLT-R IR** | **HVLT-R DR** | **L Fluency** | **Stroop C** | **Stroop R** | **Stroop I** | **Stroop IS** | **Trails A** | **Trails B** | **Trails B_Er_** | **DS** | **Coding** | **Sy Se** |
|  | **HVLT-R IR** |  | 0.71 | 0.27 | 0.16 | 0.08 | 0.10 | 0.17 | 0.24 | 0.31 | 0.15 | 0.28 | 0.27 | 0.28 |
|  | **HVLT-R DR** | 0.71 |  | 0.25 | 0.16 | 0.09 | 0.14 | 0.12 | 0.20 | 0.24 | 0.10 | 0.21 | 0.29 | 0.31 |
|  | **L Fluency** | 0.20 | 0.20 |  | 0.26 | 0.32 | 0.24 | 0.28 | 0.19 | 0.33 | 0.18 | 0.24 | 0.32 | 0.31 |
|  | **Stroop C** | 0.12 | 0.08 | 0.28 |  | 0.61 | 0.73 | 0.58 | 0.30 | 0.42 | 0.14 | 0.22 | 0.52 | 0.44 |
|  | **Stroop R** | 0.06 | -0.01 | 0.31 | 0.76 |  | 0.51 | 0.51 | 0.19 | 0.24 | 0.09 | 0.23 | 0.37 | 0.28 |
|  | **Stroop I** | 0.15 | 0.16 | 0.37 | 0.71 | 0.51 |  | 0.65 | 0.16 | 0.36 | 0.16 | 0.26 | 0.49 | 0.42 |
|  | **Stroop IS** | 0.06 | 0.06 | 0.31 | 0.52 | 0.45 | 0.62 |  | 0.24 | 0.40 | 0.10 | 0.31 | 0.52 | 0.53 |
|  | **Trails A** | 0.10 | 0.13 | 0.21 | 0.35 | 0.35 | 0.40 | 0.35 |  | 0.41 | -0.04 | 0.11 | 0.32 | 0.31 |
|  | **Trails B** | 0.13 | 0.18 | 0.28 | 0.28 | 0.21 | 0.40 | 0.40 | 0.54 |  | 0.55 | 0.28 | 0.51 | 0.47 |
|  | **Trails B_Er_** | -0.02 | 0.10 | 0.09 | 0.05 | 0.03 | 0.13 | 0.19 | 0.08 | 0.55 |  | 0.14 | 0.32 | 0.27 |
|  | **DS** | 0.20 | 0.24 | 0.26 | 0.25 | 0.18 | 0.34 | 0.18 | 0.20 | 0.36 | 0.19 |  | 0.25 | 0.25 |
|  | **Coding** | 0.12 | 0.15 | 0.27 | 0.45 | 0.39 | 0.44 | 0.53 | 0.41 | 0.44 | 0.16 | 0.27 |  | 0.60 |
|  | **Sy Se** | 0.17 | 0.13 | 0.31 | 0.44 | 0.36 | 0.46 | 0.46 | 0.42 | 0.50 | 0.17 | 0.33 | 0.64 |  |

HVLT-R=Hopkins Verbal Learning Test Revised; IR=Immediate Recall; DR=Delayed Recall; L Fluency=Verbal Fluency; Stroop=Delis-Kaplan Executive Function System (DKEFS) Color-Word Interference; C=Color Naming; R=Word Reading; I=Inhibition; IS=Inhibition/Switching; Trails A=DKEFS Trail Making Number Sequence; Trails B= DKEFS Trail Making Number Letter Sequence; DS= Wechsler Intelligence Scale for Children-V (WISC-V)/ Wechsler Adult Intelligence Scale-IV (WAIS-IV) Digit Span Backwards; Coding=WISC-V/WAIS-IV Coding; Sy Se=WISC-V/WAIS-IV Symbol Search; ER=Errors; V1=Visit 1; V2=Visit 2; V3=Visit 3; Dotted grid=pmTBI/upper diagonal; Solid grid=HC/lower diagonal. **Collinearity: white r<0.40; yellow r=0.40-0.69; red r>0.70.**

Table S7. Correlation matrices for the cognitive domain: Cogstate.

| **V1** |  | **IDN_RT_** | **IDN_ac_** | **DET_RT_** | **DET_ac_** | **OCL_RT_** | **OCL_ac_** | **ONB_RT_** | **ONB_ac_** |
| --- | --- | --- | --- | --- | --- | --- | --- | --- | --- |
|  | **IDN_RT_** |  | -0.13 | 0.71 | -0.21 | 0.53 | -0.16 | 0.65 | -0.33 |
|  | **IDN_ac_** | -0.17 |  | -0.05 | 0.50 | 0.03 | 0.36 | -0.10 | 0.37 |
|  | **DET_RT_** | 0.70 | 0.10 |  | -0.21 | 0.44 | -0.19 | 0.56 | -0.28 |
|  | **DET_ac_** | -0.23 | 0.38 | -0.11 |  | -0.04 | 0.24 | -0.16 | 0.47 |
|  | **OCL_RT_** | 0.58 | 0.01 | 0.43 | -0.06 |  | 0.08 | 0.63 | -0.10 |
|  | **OCL_ac_** | -0.20 | 0.26 | -0.14 | 0.18 | 0.03 |  | -0.18 | 0.43 |
|  | **ONB_RT_** | 0.67 | -0.09 | 0.52 | -0.16 | 0.63 | -0.17 |  | -0.41 |
|  | **ONB_ac_** | -0.25 | 0.34 | -0.18 | 0.32 | 0.04 | 0.42 | -0.40 |  |
| **V2** |  | **IDN_RT_** | **IDN_ac_** | **DET_RT_** | **DET acc** | **OCL_RT_** | **OCL acc** | **ONB_RT_** | **ONB_ac_** |
|  | **IDN_RT_** |  | -0.06 | 0.67 | -0.07 | 0.53 | -0.21 | 0.66 | -0.24 |
|  | **IDN_ac_** | -0.15 |  | 0.07 | 0.43 | 0.05 | 0.37 | -0.01 | 0.30 |
|  | **DET_RT_** | 0.74 | -0.05 |  | -0.06 | 0.36 | -0.06 | 0.52 | -0.16 |
|  | **DET_ac_** | -0.20 | 0.19 | -0.03 |  | 0.02 | 0.33 | -0.09 | 0.23 |
|  | **OCL_RT_** | 0.65 | -0.03 | 0.49 | -0.17 |  | 0.03 | 0.62 | -0.07 |
|  | **OCL_ac_** | -0.17 | 0.28 | -0.17 | 0.26 | -0.11 |  | -0.09 | 0.32 |
|  | **ONB_RT_** | 0.68 | -0.05 | 0.51 | -0.16 | 0.75 | -0.14 |  | -0.26 |
|  | **ONB_ac_** | -0.27 | 0.43 | -0.21 | 0.33 | -0.15 | 0.46 | -0.33 |  |
| **V3** |  | **IDN_RT_** | **IDN_ac_** | **DET_RT_** | **DET_ac_** | **OCL_RT_** | **OCL_ac_** | **ONB_RT_** | **ONB_ac_** |
|  | **IDN_RT_** |  | -0.07 | 0.75 | -0.21 | 0.53 | -0.19 | 0.66 | -0.38 |
|  | **IDN_ac_** | -0.32 |  | -0.04 | 0.49 | 0.02 | 0.39 | -0.07 | 0.39 |
|  | **DET_RT_** | 0.57 | -0.19 |  | -0.22 | 0.28 | -0.22 | 0.42 | -0.33 |
|  | **DET_ac_** | -0.17 | 0.42 | -0.15 |  | -0.05 | 0.33 | -0.18 | 0.48 |
|  | **OCL_RT_** | 0.70 | -0.14 | 0.34 | -0.03 |  | 0.05 | 0.70 | -0.11 |
|  | **OCL_ac_** | -0.28 | 0.49 | -0.23 | 0.31 | -0.23 |  | -0.14 | 0.41 |
|  | **ONB_RT_** | 0.70 | -0.18 | 0.40 | -0.10 | 0.76 | -0.32 |  | -0.36 |
|  | **ONB_ac_** | -0.35 | 0.47 | -0.24 | 0.27 | -0.25 | 0.48 | -0.35 |  |

IDN=Identification; DET=Detection; OCL=One-Card Learning; ONB=One-Back; RT=Reaction Time; ac=Accuracy; V1=Visit 1; V2=Visit 2; V3=Visit 3; Dotted grid=pmTBI/upper diagonal; Solid grid=HC/lower diagonal. **Collinearity: white r<0.40; yellow r=0.40-0.69; red r>0.70.**

Table S8. Correlation matrices for the retrospective and current parent ratings.

| **V1** |  | **PCS R** | **BH R** | **PA R** | **QoL R** | **PCS** | **BH** | **Symp** | **PA** | **QoL** |
| --- | --- | --- | --- | --- | --- | --- | --- | --- | --- | --- |
|  | **PCS R** |  | 0.25 | 0.44 | -0.41 | 0.30 | 0.23 | 0.41 |  |  |
|  | **BH R** | 0.37 |  | 0.50 | -0.25 | 0.02 | 0.82 | 0.31 |  |  |
|  | **PA R** | 0.60 | 0.59 |  | -0.36 | 0.13 | 0.50 | 0.44 |  |  |
|  | **QoL R** | -0.51 | -0.25 | -0.54 |  | -0.20 | -0.28 | -0.23 |  |  |
|  | **PCS** | 0.76 | 0.32 | 0.47 | -0.46 |  | 0.06 | 0.28 |  |  |
|  | **BH** | 0.32 | 0.90 | 0.54 | -0.23 | 0.33 |  | 0.34 |  |  |
|  | **Symp** | 0.32 | 0.11 | 0.25 | -0.31 | 0.38 | 0.09 |  |  |  |
|  | **PA** |  | | | | | | |  |  |
|  | **QoL** |  | | | | | | |  |  |
| **V2** |  | **PCS R** | **BH R** | **PA R** | **QoL R** | **PCS** | **BH** | **Symp** | **PA** | **QoL** |
|  | **PCS R** |  | 0.17 | 0.32 | -0.37 | 0.22 | 0.09 | 0.19 | 0.25 | -0.39 |
|  | **BH R** | 0.34 |  | 0.50 | -0.17 | 0.09 | 0.60 | 0.28 | 0.36 | -0.18 |
|  | **PA R** | 0.56 | 0.56 |  | -0.29 | 0.25 | 0.46 | 0.29 | 0.69 | -0.35 |
|  | **QoL R** | -0.49 | -0.22 | -0.52 |  | -0.20 | -0.10 | -0.16 | -0.21 | 0.43 |
|  | **PCS** | 0.46 | 0.20 | 0.34 | -0.18 |  | 0.25 | 0.34 | 0.49 | -0.46 |
|  | **BH** | 0.26 | 0.71 | 0.45 | -0.13 | 0.41 |  | 0.22 | 0.57 | -0.30 |
|  | **Symp** | 0.32 | 0.10 | 0.20 | -0.18 | 0.36 | 0.29 |  | 0.28 | -0.20 |
|  | **PA** | 0.35 | 0.37 | 0.69 | -0.38 | 0.50 | 0.55 | 0.37 |  | -0.48 |
|  | **QoL** | -0.33 | -0.27 | -0.48 | 0.50 | -0.44 | -0.35 | -0.30 | -0.63 |  |
| **V3** |  | **PCS R** | **BH R** | **PA R** | **QoL R** | **PCS** | **BH** | **Symp** | **PA** | **QoL** |
|  | **PCS R** |  | 0.16 | 0.30 | -0.38 | 0.32 | 0.08 | 0.07 | 0.23 | -0.34 |
|  | **BH R** | 0.15 |  | 0.41 | -0.12 | 0.13 | 0.45 | 0.24 | 0.27 | -0.20 |
|  | **PA R** | 0.42 | 0.48 |  | -0.26 | 0.26 | 0.31 | 0.31 | 0.60 | -0.34 |
|  | **QoL R** | -0.40 | -0.09 | -0.44 |  | -0.24 | -0.08 | -0.05 | -0.16 | 0.46 |
|  | **PCS** | 0.68 | 0.13 | 0.35 | -0.28 |  | 0.30 | 0.32 | 0.55 | -0.55 |
|  | **BH** | 0.33 | 0.53 | 0.35 | -0.08 | 0.39 |  | 0.29 | 0.55 | -0.41 |
|  | **Symp** | 0.33 | 0.11 | 0.15 | -0.20 | 0.37 | 0.22 |  | 0.38 | -0.20 |
|  | **PA** | 0.37 | 0.32 | 0.66 | -0.32 | 0.46 | 0.55 | 0.30 |  | -0.59 |
|  | **QoL** | -0.31 | -0.13 | -0.34 | 0.50 | -0.34 | -0.31 | -0.30 | -0.52 |  |

R=Retrospective Parent Report; PCS=Post-Concussion Symptoms (PCS); BH=Behavior; PA=Psychological Attributes; QoL= Quality of Life; Symp=Parent Symptoms; V1=Visit 1; V2=Visit 2; V3=Visit 3; Dotted grid=pmTBI/upper diagonal; Solid grid=HC/lower diagonal. **Collinearity: white r<0.40; yellow r=0.40-0.69; red r>0.70.**

Table S9. Variable Importance score (VIMP %) for the diagnostic and outcome models for the clinical-ratings domain: Retrospective and Current parent ratings.

| **Clinical-ratings Domain** | | | | | | | |
| --- | --- | --- | --- | --- | --- | --- | --- |
| **Metric** | **Tests** | **DX** | | | **Outcome** | | |
|  |  | **V1** | **V2** | **V3** | **V1** | **V2** | **V3** |
| Parent ratings | PCS R | 4.49* | -0.13 | 1.36 | 0.04 | 0.65 | 4.68 |
|  | Behavior R | 0.60 | 0.14 | -0.24 | 0.50 | 1.30 | -0.16 |
|  | PA R | 0.96 | 0.70 | 0.23 | 1.76 | 1.93* | 1.53 |
|  | QoL R | 4.11* | 2.54* | 2.70 | -0.22 | 2.40 | -0.16 |
|  | PCS | 34.55* | 3.80* | 3.58* | 16.68* | 9.31* | 8.43* |
|  | Behavior | 2.33* | 1.82 | 0.55 | 1.25 | 1.19 | -0.37 |
|  | Symptoms | 1.67 | 0.23 | 0.51 | 0.19 | 3.97* | 2.46 |
|  | PA | NA | 2.24 | 1.50 | NA | 4.22* | 3.52* |
|  | QoL | NA | 4.23* | 0.47 | NA | -0.62 | 1.47 |
| ROC Curve | AUC | 0.85 | 0.68 | 0.64 | 0.67 | 0.68 | 0.66 |
|  | Balanced Accuracy | 0.79 | 0.66 | 0.62 | 0.66 | 0.68 | 0.66 |
|  | Sensitivity | 0.76 | 0.69 | 0.55 | 0.63 | 0.73 | 0.64 |
|  | Specificity | 0.83 | 0.63 | 0.69 | 0.69 | 0.62 | 0.67 |

V1=Visit 1 (~7 days post-injury); V2=Visit 2 (~4-months post-injury); V3=Visit 3 (~1-year post-injury); Diagnostic (DX); R=Retrospective Parent ratings; PCS=Post-Concussion Symptoms; PA=Psychological Attributes; QoL=Quality of Life; Symptoms=Parent Symptoms; ROC=Receiver operating characteristic; AUC=overall performance; * denotes features that were selected in the individual domain models.

**References**

Beers, S. R., Wisniewski, S. R., Garcia-Filion, P., Tian, Y., Hahner, T., Berger, R. P., . . . Adelson, P. D. (2012). Validity of a pediatric version of the Glasgow Outcome Scale-Extended [10.1089/neu.2011.2272 doi]. *J.Neurotrauma*, *29*(6), 1126-1139. PM:22220819

Buysse, D. J., Yu, L., Moul, D. E., Germain, A., Stover, A., Dodds, N. E., . . . Pilkonis, P. A. (2010). Development and validation of patient-reported outcome measures for sleep disturbance and sleep-related impairments. *Sleep*, *33*(6), 781-792. PM:20550019

Delis, D. C., Kaplan, E., & Kramer, J. H. (2001). *Delis-Kaplan executive function system (D-KEFS)*. Psychological Corporation.

Derogatis, L. R., & Fitzpatrick, M. (2004). The SCL-90-R, the Brief Symptom Inventory (BSI), and the BSI-18.

Farrar, J. T., Young, J. P., Jr., LaMoreaux, L., Werth, J. L., & Poole, R. M. (2001). Clinical importance of changes in chronic pain intensity measured on an 11-point numerical pain rating scale [S0304-3959(01)00349-9 pii]. *Pain*, *94*(2), 149-158. PM:11690728

Gioia, G. A., Collins, M., & Isquith, P. K. (2008). Improving identification and diagnosis of mild traumatic brain injury with evidence: psychometric support for the acute concussion evaluation [10.1097/01.HTR.0000327255.38881.ca doi ;00001199-200807000-00005 pii]. *J.Head Trauma Rehabil.*, *23*(4), 230-242. <https://doi.org/10.1097/01.HTR.0000327255.38881.ca>

Gioia, G. A., Schneider, J. C., Vaughan, C. G., & Isquith, P. K. (2009). Which symptom assessments and approaches are uniquely appropriate for paediatric concussion? [43/Suppl_1/i13 pii ;10.1136/bjsm.2009.058255 doi]. *Br.J.Sports Med.*, *43 Suppl 1*, i13-i22. <https://doi.org/10.1136/bjsm.2009.058255>

Goodman, R. (1997). The Strengths and Difficulties Questionnaire: a research note. *J.Child Psychol.Psychiatry*, *38*(5), 581-586. PM:9255702

Hergert, D. C., Sicard, V., Stephenson, D. D., Pabbathi, R. S., Robertson-Benta, C. R., Dodd, A. B., . . . Mayer, A. R. (2022). Test-Retest Reliability of a Semi-Structured Interview to Aid in Pediatric Traumatic Brain Injury Diagnosis [S1355617721000928 pii ;10.1017/S1355617721000928 doi]. *J Int.Neuropsychol.Soc.*, *28*(7), 687-699. <https://doi.org/10.1017/S1355617721000928>

Kosinski, M., Bayliss, M. S., Bjorner, J. B., Ware, J. E., Jr., Garber, W. H., Batenhorst, A., . . . Tepper, S. (2003). A six-item short-form survey for measuring headache impact: the HIT-6. *Qual.Life Res.*, *12*(8), 963-974. PM:14651415

Kriz, P. K., Stein, C., Kent, J., Ruggieri, D., Dolan, E., O'Brien, M., & Meehan, W. P., III. (2016). Physical Maturity and Concussion Symptom Duration among Adolescent Ice Hockey Players [S0022-3476(15)01516-4 pii ;10.1016/j.jpeds.2015.12.006 doi]. *J.Pediatr.*, *171*, 234-239. PM:26781190

Mayer, A. R., Stephenson, D. D., Dodd, A. B., Robertson-Benta, C. R., Pabbathi, R. S., Shaff, N. A., . . . Quinn, D. K. (2020). Comparison of Methods for Classifying Persistent Post-Concussive Symptoms in Children [10.1089/neu.2019.6805 doi]. *J Neurotrauma*, *37*(13), 1504-1511. <https://doi.org/10.1089/neu.2019.6805>

Pilkonis, P. A., Choi, S. W., Reise, S. P., Stover, A. M., Riley, W. T., & Cella, D. (2011). Item banks for measuring emotional distress from the Patient-Reported Outcomes Measurement Information System (PROMIS(R)): depression, anxiety, and anger [1073191111411667 pii ;10.1177/1073191111411667 doi]. *Assessment.*, *18*(3), 263-283. PM:21697139

Prinz, R. J., Foster, S., Kent, R. N., & O'Leary, K. D. (1979). Multivariate assessment of conflict in distressed and nondistressed mother-adolescent dyads [10.1901/jaba.1979.12-691 doi]. *J.Appl.Behav.Anal.*, *12*(4), 691-700. PM:541311

Sady, M. D., Vaughan, C. G., & Gioia, G. A. (2014). Psychometric characteristics of the postconcussion symptom inventory in children and adolescents [acu014 pii ;10.1093/arclin/acu014 doi]. *Arch.Clin.Neuropsychol.*, *29*(4), 348-363. <https://doi.org/10.1093/arclin/acu014>

Varni, J. W., Seid, M., & Rode, C. A. (1999). The PedsQL: measurement model for the pediatric quality of life inventory. *Med.Care*, *37*(2), 126-139. PM:10024117

Wechsler, D. (2008). *Wechsler adult intelligence scale-fourth*. San Antonio: Pearson.

Wechsler, D. (2014). Wechsler intelligence scale for children - Fifth edition (WISC-V): Technical and interpretive manual. In: Bloomington, MN: Pearson Clinical Assessment.

Zemek, R., Barrowman, N., Freedman, S. B., Gravel, J., Gagnon, I., McGahern, C., . . . Osmond, M. H. (2016). Clinical risk score for persistent postconcussion symptoms among children with acute concussion in the ED [2499274 pii ;10.1001/jama.2016.1203 doi]. *JAMA*, *315*(10), 1014-1025. <https://doi.org/10.1001/jama.2016.1203>
